# NV Proteins of Fish Novirhabdovirus Recruit Cellular PPM1Bb Protein Phosphatase and Antagonize RIG-I-Mediated IFN Induction

**DOI:** 10.1038/srep44025

**Published:** 2017-03-09

**Authors:** Stéphane Biacchesi, Emilie Mérour, Didier Chevret, Annie Lamoureux, Julie Bernard, Michel Brémont

**Affiliations:** 1 VIM, INRA, Université Paris-Saclay, 78350, Jouy-en-Josas, France; 2 PAPPSO, Micalis Institute, INRA, AgroParisTech, Université Paris-Saclay, 78350, Jouy-en-Josas, France

## Abstract

Non virion (NV) protein expression is critical for fish Novirhabdovirus, viral hemorrhagic septicemia virus (VHSV) and infectious hematopoietic necrosis virus (IHNV), *in vivo* pathogenesis. However, the mechanism by which NV promotes the viral replication is still unclear. We developed an approach based on reverse genetics and interactomic and identified several NV-associated cellular partners underlying cellular pathways as potential viral targets. Among these cell partners, we showed that NV proteins specifically interact with a protein phosphatase, Mg^2+^/Mn^2+^-dependent, 1Bb (PPM1Bb) and recruit it in the close vicinity of mitochondria, a subcellular compartment important for retinoic acid-inducible gene-I- (RIG-I)-mediated interferon induction pathway. PPM1B proteins belong to the PP2C family of serine/threonine (Ser/Thr) protein phosphatase and have recently been shown to negatively regulate the host antiviral response via dephosphorylating Traf family member-associated NF-κB activator (TANK)-binding kinase 1 (TBK1). We demonstrated that NV proteins and PPM1Bb counteract RIG-I- and TBK1-dependent interferon (IFN) and IFN-stimulated gene promoter induction in fish cells and, hence, the establishment of an antiviral state. Furthermore, the expression of VHSV NV strongly reduced TBK1 phosphorylation and thus its activation. Our findings provide evidence for a previously undescribed mechanism by which a viral protein recruits PPM1Bb protein phosphatase to subvert innate immune recognition.

Viral hemorrhagic septicemia virus (VHSV) and infectious hematopoietic necrosis virus (IHNV) are causative agents of very contagious and acute systemic diseases leading to high mortality mainly, but not exclusively, in young salmonids worldwide. Both viruses are listed as notifiable by the World Organisation for Animal Health (OIE)^1^. VHSV and IHNV are considered as serious economic and social threats for fish farms with significant environmental impact on natural resources^2^,^3^. For example, VHSV was the cause of mass mortalities involving at least 31 freshwater fish species in the 2000 s in the Great Lakes region of the Northern America^4^. IHNV and VHSV belong to the family *Rhabdoviridae* within the order *Mononegavirales*^5^,^6^. These viruses are enveloped and their genomes consist of a non-segmented negative-sense single-stranded RNA molecule of about 11 kilobases which encode six proteins in the order 3′-N-P-M-G-NV-L-5′^7^,^8^,^9^. The viral RNA, which encodes five structural proteins, is tightly encapsidated with a nucleoprotein (N), a polymerase-associated phosphoprotein (P) and the large RNA-dependent RNA polymerase (L) to form the helical ribonucleoprotein complex (RNP). The matrix protein (M) interacts with the RNP and the viral envelope where is inserted the unique viral surface glycoprotein (G)^10^.

In contrast to other rhabdoviruses, IHNV and VHSV genomes possess an additional gene, localized between the G and L genes, that encodes a small non-structural NV protein (Non Virion protein of 111 and 122 amino acids, respectively)^11^,^12^,^13^. Due to the presence of the NV gene, IHNV and VHSV were classified together in the genus *Novirhabdovirus*^6^. The NV protein is expressed at low levels in infected cells and is localized both in the cytoplasm and nucleus^12^,^13^,^14^,^15^. The recovery of NV-deletion mutants, by reverse genetics^16^, demonstrated that the NV protein was dispensable but necessary for an efficient virus replication in cell culture^17^,^18^,^19^,^20^ and essential for virus pathogenicity in rainbow trout^21^,^22^, yellow perch^17^ and Japanese olive flounder^20^. Using recombinant IHNV expressing the luciferase gene and a non-invasive bioluminescence imaging device, it was further shown that the NV-deletion leads to a very limited virus propagation in the host. The deletion mutant was blocked in the fins, the virus portal entry into rainbow trout, and could persist there for a relatively long period^21^. Interestingly, the IHNV and VHSV NV proteins, which exhibit a low percentage of homology (31%) and no obvious conserved domains, share a similar function. Indeed, a recombinant IHNV, rIHNV-NVvhsv, in which the IHNV NV gene was replaced by that of VHSV, was shown to replicate efficiently in fish cells and to induce similar cumulative percentage of mortality *in vivo* in rainbow trout compared to the wild-type (WT) IHNV^22^. Comparative studies between WT and NV-deletion mutant IHNV or VHSV suggest that NV proteins may downregulate the induction of interferon (IFN) and interferon-stimulated genes (ISG) during *in vitro* infection of rainbow trout- or cyprinid-derived cell lines^15^,^23^ and *in vivo* infection of olive flounder^23^. Moreover, the inhibition of virus-induced IFN response by NV depends on its localization in the infected-cell nucleus^15^. Other recent evidences also propose that VHSV NV protein suppresses TNFα-mediated NF-κB activation^24^ and has an anti-apoptotic function early in infection *in vitro*^25^. Finally, Chinchilla *et al*. showed an extensive downregulation of several innate and adaptive immune-related genes in head kidney and spleen of rainbow trout after injection of recombinant VHSV NV protein, indicating its potent immunosuppressive activity on antiviral immune responses^26^. However, the mechanisms by which NV proteins interfere with the host responses remains unsolved.

The IFN system is remarkably conserved in vertebrates and teleost fish possess functional orthologs of human retinoic acid-inducible gene-I (RIG-I)-like receptors (RLRs), including RIG-I, MDA5 and LGP2, as well as several downstream signaling molecules, such as MAVS, TBK1 and IRF3^27^,^28^,^29^. The pathogen components, such as nucleic acids and viral proteins, are recognized through specific pattern-recognition receptors (PRRs), leading to the activation of multiple signaling cascades that induce the production of interferons (IFN) and other cytokines. Among the PRRs, RLRs play a key role in sensing viral RNA in the cell cytosol and are essential in the early induction of type I IFN^30^. Activation of RIG-I leads to its interaction with the mitochondrial activator of virus signaling (MAVS) protein, the essential signaling adapter protein of the RLRs located at the surface of mitochondria. This interaction induces the recruitment of downstream molecules and the activation of TANK-binding kinase 1 (TBK1) and inhibitor-κB kinase ε (IKKε) protein kinases which activate the transcription factors interferon regulatory factor (IRF) 3 and NF-κB through phosphorylation steps. Upon activation, IRF3 and NF-κB translocate from the cytosol to the nucleus to induce the expression of the type I IFNs and inflammatory cytokines. Because of the important role of this pathway in the early interferon expression, viruses have evolved multiple strategies to evade host RLR mediated signaling^31^,^32^.

Here, we report the establishment of an original system where triple flag-tagged NV proteins from IHNV and VHSV are overexpressed during the viral infection. These recovered recombinant viruses have similar growth properties *in vitro* in cell culture and *in vivo* in rainbow trout compared to those of the WT virus, and thus they have been used to co-purify NV and its associated proteins from infected cells. Purified protein complexes were subjected to multidimensional liquid chromatography (LC) and tandem mass spectrometry (MS/MS) analysis. We found that NV associated with several host proteins, in particular, a member of the PP2C family of serine/threonine (Ser/Thr) protein phosphatase, the phosphatase, Mg^2+^/Mn^2+^-dependent, 1Bb (PPM1Bb). Mammalian PPM1B have been recently identified as a negative regulator of the antiviral response via TBK1 dephosphorylation^33^,^34^. TBK1 mediates the activation of interferon regulatory factor (IRF) 3, leading to the induction of type I IFN following viral infection. We verified that NV proteins specifically interact with PPM1Bb and observed that NV proteins re-localize PPM1Bb in close proximity to the mitochondrial network. We demonstrated that the expression of NV or PPM1Bb negatively regulates antiviral signaling by targeting TBK1, indicating the important role of PPM1Bb for IHNV and VHSV to evade innate immunity.

## Results

### Recovery of recombinant VHSV (rVHSV) overexpressing tagged-NV proteins

**Figure 1:**
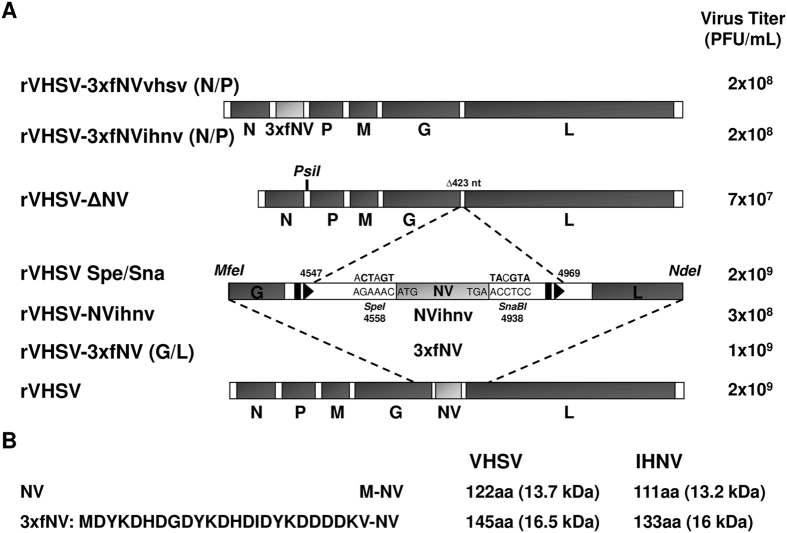
Construction of the NV-gene VHSV mutants. (**A**) NV-gene VHSV mutants. The wild-type VHSV genome is shown at the bottom, drawn approximately to scale. The enlarged diagram shows the MfeI/NdeI fragment containing the NV gene. The NV ORF is shown as a rectangle flanked on the upstream and downstream ends by the gene start (black triangle) and gene end (black rectangle) transcription signals, respectively. Also shown are SpeI and SnaBI unique restriction sites that were introduced into the non-coding regions surrounding the NV ORF: the sequence and nucleotide position of each site (based on the position of its first residue in the antigenomic sequence) are shown, with nucleotide substitutions made to create the sites in bold and the wild-type sequence shown underneath. This construct, called pVHSV Spe/Sna, was used to generate rVHSV-NVihnv and rVHSV-3xfNV viruses encoding the NV from IHNV and a VHSV NV protein fused to a triple Flag tag (3xfNV), respectively. The NV gene-deletion mutant (rVHSV-∆NV) was created by removing the entire NV region including the transcription signals, the NV ORF and the surrounding non-coding regions (deletion of 423 nucleotides). Two other viruses were constructed by the insertion in the intergenic region between the N and P genes of an additional expression cassette encoding the VHSV and IHNV 3xfNV proteins in the rVHSV-∆NV genome leading to rVHSV-3xfNVvhsv (N/P) and rVHSV-3xfNVihnv (N/P), respectively. Titers of each recombinant virus are indicated to the left in PFU/mL. (**B**) The amino acid sequence of the triple Flag tag fused to the N-terminal end of the NV proteins. The lengths and the molecular weights of the wild-type NV and the tagged NV (3xfNV) are indicated to the left.

In an effort to identify cellular partners of NV proteins, we first designed, using reverse genetics, VHSV mutants in which NV from both Novirhabdoviruses were overexpressed with an N-terminal tag allowing then an easier immunopurification of the proteins. We took advantage of the gradient of expression found in *Mononegavirales* in which the 3′ proximal genes are more transcribed than those located at the 5′ end^35^,^36^. The previously described expression cassette located between N and P genes was used to this purpose^18^. By mutagenesis, the complete gene encoding VHSV NV was deleted together with the gene start and gene end signals, leading to rVHSV-∆NV (Fig. 1A). In parallel, a triple flag tag was fused to the N terminus of both NV proteins (Fig. 1B). The mutated genes were then inserted either at the original NV position between G and L genes, rVHSV-3xfNV (G/L), or in the expression cassette inserted between N and P genes, rVHSV-3xfNV (N/P) (Fig. 1A).

**Figure 2:**
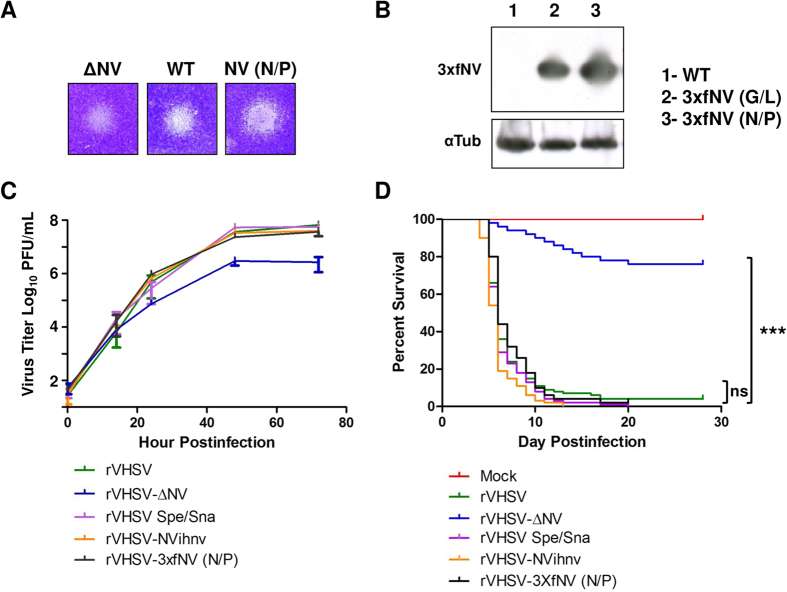
*In vitro* and *in vivo* characterization of rVHSV overexpressing tagged-NV protein. (**A**) Plaque phenotypes. EPC cells were infected with approximately 10 PFU of rVHSV-∆NV, wild-type rVHSV, rVHSV-3xfNV (N/P) and cultured in an agarose-containing medium. At 5 days postinfection, cell monolayers were fixed and stained with crystal violet. (**B**) Viral overexpression of the NV protein. Western-blot analysis was performed on lysates from wild-type rVHSV (lane 1), rVHSV-3xfNV (G/L) (lane 2) and rVHSV-3xfNV (N/P) (lane 3) infected cells to analyze the NV expression. Full-length blots are included in the Fig. S7. (**C**) Multi-step growth curves. EPC cells were infected at an MOI of 0.01 PFU per cell with wild-type rVHSV, rVHSV-∆NV, rVHSV Spe/Sna, rVHSV-NVihnv and rVHSV-3xfNV (N/P). Supernatant aliquots were taken on the indicated days postinfection and analyzed later by plaque assay. Each time point was represented by two wells, and each virus titration was done in duplicate. Means are shown together with the calculated standard errors. (**D**) Percentage of survival in trout infected by the VHSV NV-gene mutants. Juvenile rainbow trout (100 fish/tank; mean weight, 1.5 g) were infected by bath immersion with 5 × 10^4 ^PFU/mL of wild-type rVHSV, rVHSV-∆NV, rVHSV Spe/Sna, rVHSV-NVihnv, rVHSV-3xfNV (N/P) or mock infected as a negative control. Mortalities were recorded every day for 28 days. For statistical analysis, a comparison of survival between groups was performed with the log rank test on the Kaplan-Meier survival data using GraphPad Prism (GraphPad, San Diego, CA). Groups that are not significantly different from each other are noted ns (P > 0.05), whereas those that are significantly different are noted ***(P < 0.001).

rVHSVs were readily recovered by transfection of vTF7-3-infected EPC cells with each respective full-length plasmid together with the three support plasmids encoding the VHSV N, P and L proteins^18^. The recombinant viruses were amplified by two passages in EPC cells, and two-passage stocks were used in all experiments. Viral RNA from each VHSV mutant was subjected to reverse transcription-PCR using specific primers. In each case, the product obtained was consistent with the expected deletion and/or insertion (data not shown). Furthermore, each fragment was completely sequenced, confirming the expected modified genome structure. Each VHSV mutant reached almost similar titers, between 2 × 10^8^ and 2 × 10^9 ^PFU/mL, with the exception of rVHSV-∆NV which had a lower titer of 7 × 10^7 ^PFU/mL (Fig. 1A). Virus induced plaque phenotypes were slightly different (Fig. 2A) and correlated to the amount of expressed NV by the different mutants (Fig. 2B). Indeed, the shift of the NV gene along the VHSV genome does increase the expression of the NV protein in infected cells together with the cytopathic effect.

The mutants were then compared to wild-type rVHSV (rVHSVwt) with regard to the efficiency of multicycle replication in EPC cells following infection with an input of 0.01 PFU per cell (Fig. 2C). As previously shown, rVHSV-∆NV replicated with a lower efficiency *in vitro* compared to rVHSVwt (10- to 100-fold lower)^17^,^18^ and both NV proteins could efficiently complement each other^22^,^25^. Indeed, rVHSVwt and rVHSV-NVihnv, in which the VHSV NV gene was replaced by that of IHNV, replicated with similar efficiencies *in vitro*. Interestingly, the addition of a triple flag tag at the N-terminus and/or the increase of the expression of both NV proteins did not lead to any adverse effect on the virus replication. rVHSV expressing tagged-NV proteins from N/P positions, rVHSV-3xfNV (N/P), replicated as well as rVHSVwt in EPC cells.

Finally, *in vivo* experiments were performed to make sure that the overexpression of tagged-NV did not lead to any particular phenotype. Juvenile rainbow trout (100 fish per group) were infected by bath immersion with 5 × 10^4 ^PFU/mL of each virus. rVHSV-∆NV was highly attenuated *in vivo* and only induced 24% of cumulative mortality compared to almost 100% for rVHSVwt. In contrast, the recombinant virus that overexpressed tagged NV proteins was still highly virulent and undistinguishable to rVHSVwt, minimizing the possibility of missing any NV function related to the tag fusion. Thus, these viruses seemed to be useful overexpressing vectors to identify cellular partners of NV proteins in an infectious context.

### Identification of host proteins associated with VHSV and IHNV NV proteins

**Figure 3:**
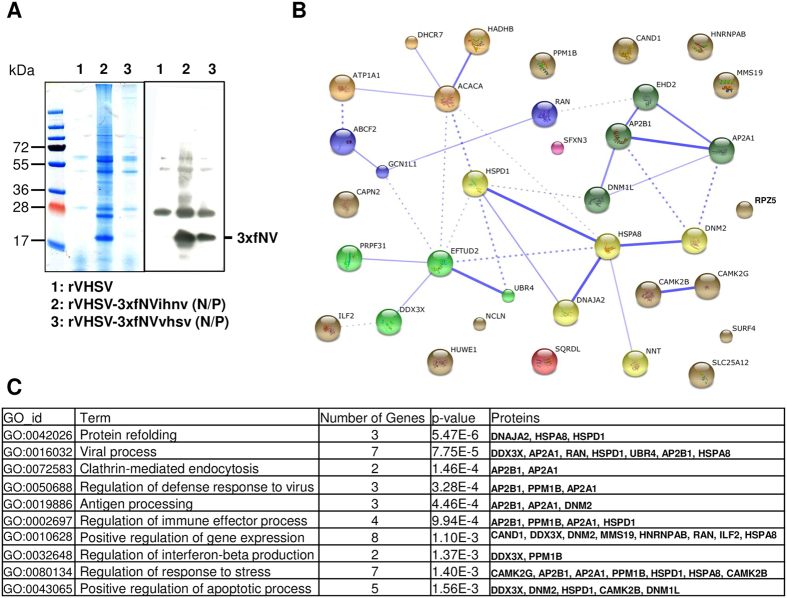
Identification of host proteins that co-precipitated with VHSV and IHNV NV proteins. (**A**) Co-immunoprecipitation of cellular proteins associated with NV proteins. Lysates from rVHSV (background control; lane 1), rVHSV-3xfNVihnv (N/P) (lane 2) and rVHSV-3xfNVvhsv (N/P) (lane 3) infected cells were immunoprecipitated using anti-Flag mAb. Aliquot of each eluate was analyzed by electrophoresis on a polyacrylamide gel followed by Coomassie blue staining (left panel) or western-blotting using the anti-Flag mAb (right panel). (**B**) NV-associated host protein network. The protein-protein interaction network involving the 35 host proteins associated with NV were shown in the confidence view produced by STRING v10 analysis. Stronger associations are represented by thicker lines. (**C**) Gene ontology (GO biological processes) enrichment analysis of host proteins co-immunoprecipitating with NV proteins.

The foregoing results showed that both rVHSV-3xfNVvhsv (N/P) and rVHSV-3xfNVihnv (N/P) mutants were suitable for use in experiments to identify the cellular proteins that associate with NV during virus infection. EPC cells were infected with each mutant (multiplicity of infection (MOI) of 1) and a control was performed with rVHSVwt which does not express any triple flag tagged protein. At 48 h postinfection, cell lysates were used to immunoprecipitate NV proteins using an anti-flag tag mAb (see materials and methods). The purified protein complexes were analyzed on a polyacrylamide gel (Fig. 3A, left) and the presence of the NV proteins was confirmed by western-blot assay (Fig. 3A, right). Finally, the protein complexes were subjected directly to protein identification by LC-MS/MS.

The LC-MS/MS analysis identified that NV associates with the other viral proteins (Table S1A). An additional experiment confirmed this first observation (Fig. S1). Indeed, NV was co-purified by immunoprecipitation of the N protein from infected cell lysates. A list of cellular proteins was additionally identified. To reduce the list to cellular proteins that may directly associate with NV alone, another LC-MS/MS analysis was performed using NV protein complexes immunoprecipited from EPC cells transfected with DNA plasmid vectors expressing VHSV or IHNV triple flag-tagged NV proteins. After compilation of all the results, only the cellular proteins that were detected at least once for both NV proteins and in both analyses were conserved. Finally, NV proteins associate at least with 35 host proteins (Table S1B1 and Fig. 3B). These proteins are located in both cytoplasm (24 proteins) and nucleus (15 proteins) where NV was also previously localized^15^. The most abundant proteins are RNA-binding proteins (11 proteins) that are involved in RNA processing, RNA transcription, mRNA localization and regulation of gene expression (Fig. 3C). Several of these proteins have already been associated with viral processes such as viral genome replication or intracellular transport of virus (7 proteins) and host defense against virus infection such as antigen processing (3 proteins), regulation of stress response (8 proteins), regulation of IFNβ production (2 proteins) and apoptotic process (5 proteins). Finally, cellular proteins found in both kinds of analysis and unique for IHNV or VHSV NV proteins are presented in Tables S1B2 and S1B3, respectively.

Among the identified cellular partners of NV, we were particularly interested in proteins involved in the regulation of interferon production. Indeed, as shown in Fig. S2, NV expression is required for the efficient inhibition of virus-induced IFN, as previously observed^15^,^23^. By analyzing the LC-MS/MS data, we found that three proteins, DDX3 (a DEAD-box RNA helicase^37^), EFTUD2 (a spliceosomal GTPase^38^) and PPM1Bb (a serine/threonine protein phosphatase type 2 C (PP2C)^34^) were previously described in mammals as regulators of type I IFN production (Fig. 3C). In order to validate this list of potential NV partners, the interactions between NV and EFTUD2, DDX3 and PPM1Bb were confirmed by immunoprecipitation and Western-blot analysis on lysates from co-transfected EPC cells (Fig. S3A). Given the recent findings on PP2C negative regulation of antiviral response via dephosphorylating TBK1^34^,^39^; we decided to focus on the potential role of the interaction between NV and PPM1Bb in Novirhabdovirus infection.

### Identification of PPM1Bb as a NV-interacting protein

In order to validate the specific interaction of PPM1Bb with NV, we first cloned the full-length PP2C family member-related cDNA of cyprinid EPC cells and salmonid TO (from Atlantic salmon) and RTG-2 (from rainbow trout) cells. We focused on both PPM1A and PPM1B since these two PP2C proteins (also known as PP2Cα and PP2Cβ, respectively), which are highly similar at the amino acid level, were described as TBK1 phosphatases^34^,^39^. The two peptides detected in LC-MS/MS analysis were specific to the fish PPM1Bb paralog, thus we focused on this PPM1Bb paralog and its closest related isoenzyme PPM1Aa. Specific primers (Table S2) were designed as described in Materials and Methods and used to amplify cDNA molecules from EPC, RTG-2 and TO cells, respectively. These PP2C members were readily amplified from each cell line and fully sequenced (accession numbers in Table S3). The deduced protein sequences were subjected to multiple alignments with PP2C proteins from mammalian and other fish species, showing that PPM1A and PPM1B were highly conserved during the evolution (Table S4A and B). The phylogenetic analysis also suggested that all these sequences constitute orthologs (Fig. S4A). Finally, we confirmed that identified fish sequences of PPM1Aa and Bb were highly similar at the amino acid level (Table S4C). Thus, we searched for conserved synteny involving ppm1a and b genes and other markers in the neighborhood. At least two copies of ppm1a and ppm1b genes, named ppm1aa or ppm1ab and ppm1ba or ppm1bb, respectively, were found in the zebrafish genome. Similarly, at least two copies of each gene were also found in other fish genomes (Fig. S4A). We could identify several markers that are conservatively found in the genomic region where both ppm1aa and ppm1ab (Fig. S4B) and ppm1ba and ppm1bb (Fig. S4C) paralogs are located in zebrafish and mammals, reinforcing the idea that they are all true orthologs.

**Figure 4:**
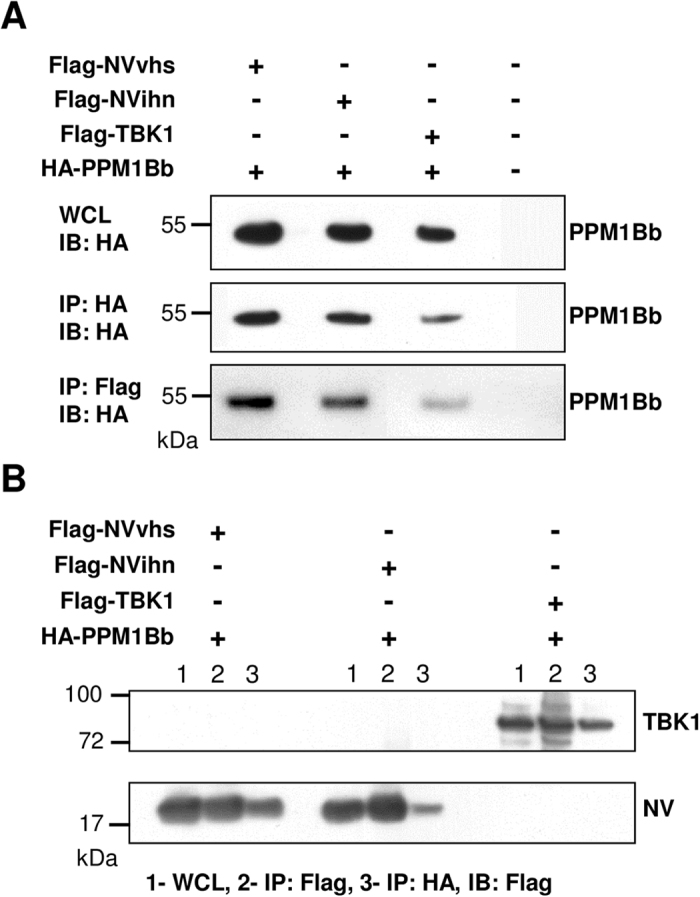
Identification of PPM1Bb as a protein that interacts with both NV proteins. Epitope-tagged PPM1Bb and NV proteins interact with each other in EPC cells. EPC cells were co-transfected with 1 μg of the indicated plasmids. At 48 h post transfection, the lysates were immunoprecipitated (IP) with anti-HA or anti-Flag as indicated, followed by immunoblotting (IB) analysis with anti-HA (**A**) or anti-Flag (**B**). WCL corresponds to the expression of exogenous proteins in whole-cell lysates. TBK1 (84 kDa) was added as a positive interactor for PPM1Bb. Full-length blots are included in the Fig. S7.

To confirm that PPM1Bb interacts physically with NV proteins, we performed co-immunoprecipitation experiments. TBK1 from EPC cells was characterized and used as a positive interacting partner of PPM1B^34^. As shown in Fig. 4A and B, epitope-tagged PPM1Bb and NV originated from both VHSV and IHNV reciprocally co-immunoprecipitated with each other in transfected EPC cells as well as TBK1 interacts with PPM1Bb, demonstrating that PPM1Bb is a cellular partner of NV proteins. Using different tags, this specific interaction between NV and PPM1Bb was confirmed (Fig. S3B). Moreover, this interaction was highly specific since PPM1Aa, which shares 75% of identity with PPM1Bb (Table S4C), does not interact with NV (Fig. S5). Finally, although VHSV infects a broad range of freshwater and marine fish species, IHNV has a more restricted tropism and affects mainly salmonid fish. Therefore, we controlled that IHNV NV could interact with PPM1Bb originated from a salmon cell line. As shown in Fig. S5, Salmon PPM1Bb co-immunoprecipitates with IHNV NV, confirming the interest in understanding this particular interaction.

### NV proteins recruit PPM1Bb in close vicinity to mitochondria

**Figure 5:**
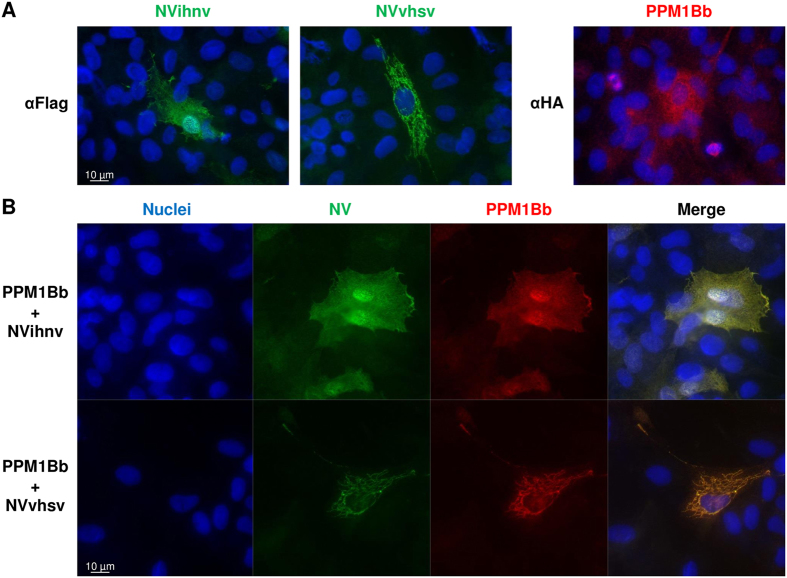
PPM1Bb is co-localized with both NV proteins in transfected EPC cells. EPC cells were transfected with 1.5 μg of the indicated plasmids alone (**A**) and in combination (**B**) for 48 h, and then fixed. PPM1Bb and NV proteins were detected with anti-HA and anti-Flag mAbs, respectively. The nuclei were stained with DAPI (blue).

**Figure 6:**
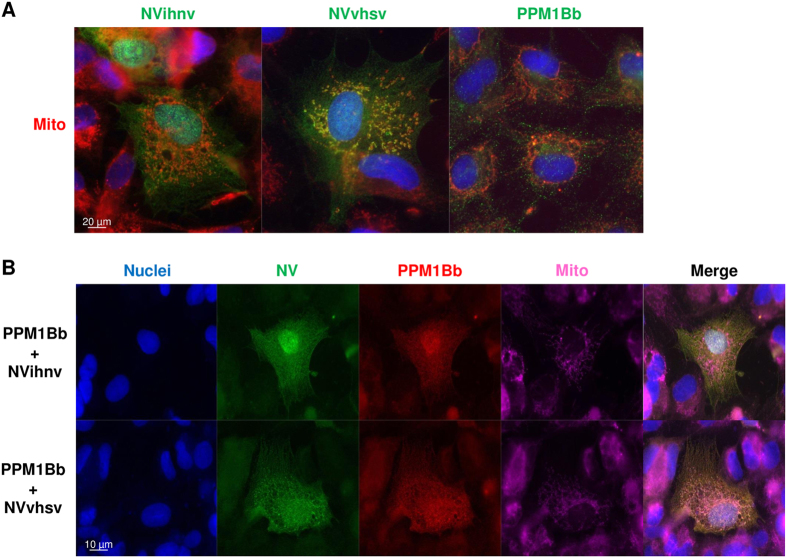
PPM1Bb is co-localized with both NV proteins in close proximity to mitochondria. EPC cells were transfected with 1.5 μg of the indicated plasmids alone (**A**) and in combination (**B**) for 24 h. Cell mitochondria were then stained with a red MitoTracker. After fixation and permeabilization, PPM1Bb and NV proteins were detected with anti-HA and anti-Flag mAbs, respectively. The nuclei were stained with DAPI (blue).

As previously described^15^, we confirmed by immunostaining assays that IHNV NV protein was localized both in the cytoplasm and nucleus (Fig. 5A). In contrast, VHSV NV protein preferentially localized to the cytoplasm of EPC cells, with a minor proportion in the nucleus. This localization of NV proteins was confirmed in infected EPC cells (data not shown). PPM1Bb was also predominantly found in the cytoplasm and to a lesser extent in the nucleus of EPC cells, as observed in mammalian cells^40^. Interestingly, PPM1Bb was observed tightly linked to the mitotic spindles of dividing cells (Fig. 5A), a similar location where high levels of active phospho-TBK1 was recently described during cell mitosis^41^. When both proteins were expressed together, however, PPM1Bb re-localized to the cytoplasm, where it extensively co-localized with NV (Fig. 5B). The re-localization of PPM1Bb was particularly pronounced with VHSV NV protein. Remarkably, PPM1Bb was also found in the nucleus where it co-localized with IHNV NV. Previous studies showed that phospho-TBK1, one of the various substrates of mammalian PPM1B phosphatases^34^, was localized at mitochondria-associated membranes^42^,^43^, a subcellular compartment that links the endoplasmic reticulum to the mitochondria and where MAVS and STING adaptors recruit TBK1^33^. As shown in Fig. 6A, IHNV NV seems to be localized in close vicinity to the mitochondria network and the co-localization was even more pronounced for VHSV NV. In contrast, no co-localization could be detected between PPM1Bb and the mitochondria. However, when PPM1Bb was co-expressed with NV proteins, the phosphatase was re-localized in close proximity to the mitochondria network (Fig. 6B). On the basis of these observations, we reasoned that NV proteins might recruit PPM1Bb to inhibit TBK1 activity and, hence the establishment of an antiviral state.

### Overexpression of fish PPM1Bb or NV proteins inhibits RIG-I-mediated antiviral signaling

**Figure 7:**
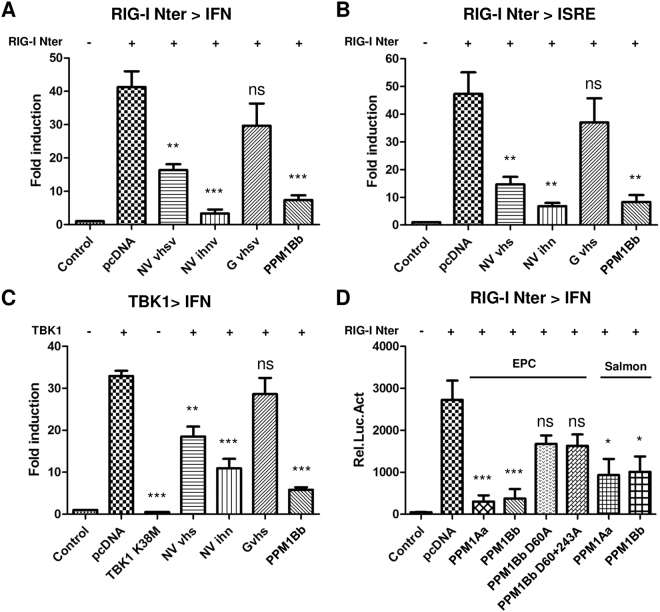
Overexpression of fish PPM1Aa and PPM1Bb or NV proteins inhibits RIG-I-mediated antiviral signaling. (**A**,**B**) Overexpression of PPM1Bb or NV proteins reduced RIG-I-induced activation of the IFN1 and ISRE promoters. EPC cells were transfected with the indicated plasmids (1 μg) together with luciferase reporter constructs (1 μg) driven by the promoters of genes encoding IFN1 (**A**) or ISRE (**B**), and RIG-I Nter-eGFP as inducer and internal control (1 μg). In cases where RIG-I Nter-eGFP was omitted peGFP (1 μg) was added as internal control. All transfection mixtures were adjusted with and empty vector to contain an equal quantity of DNA plasmid. Twenty-four hours after transfection, the cells were lysed for luciferase assays. Luciferase activity was measured and normalized to eGFP fluorescence. The fold inductions were calculated as the ratio of stimulated (+RIG-I-Nter) versus unstimulated (Control; −RIG-I-Nter) samples. Means of at least three independent experiments are shown together with the standard errors. For statistical analysis, a comparison between groups was performed with a one-way ANOVA and Tukey’s multiple comparison tests using GraphPad Prism (GraphPad, San Diego, CA). Groups that are not significantly different from each other are noted ns (P > 0.05), whereas those that are significantly different are noted *(P < 0.05), **(P < 0.01) or ***(P < 0.001). (**C**) Overexpression of PPM1Bb or NV proteins reduced TBK1-induced activation of the IFN1 promoters. EPC cells were transfected with the indicated plasmids (1 μg) together with a luciferase reporter construct driven by the promoter of IFN1 (1 μg), TBK1 (1 μg) as inducer and peGFP (1 μg) as internal control. Twenty-four hours post transfection, luciferase assays were performed and analyzed as described above. (**D**) Fish PPM1Aa and Bb regulate RIG-I-mediated IFN expression. EPC cells were transfected with a luciferase reporter construct driven by the promoter of IFN1 (1 μg), and RIG-I Nter-eGFP as inducer and internal control (1 μg) together with DNA plasmids (1 μg) encoding wild-type or mutant forms of PPM1Aa or Bb proteins from fathead minnow and salmon, as indicated. Twenty-four hours post transfection, luciferase assays were performed, and relative luciferase activity were calculated and analyzed as described above.

To determine whether PPM1Bb and NV proteins are both involved in the inhibition of the cellular antiviral response, we first tested whether those proteins contribute to the inhibition of RIG-I-mediated type I IFN signaling by using a cell-based luciferase reporter system. In all conditions tested below, promoter activities were normalized to the levels of eGFP fluorescence expressed alone or in fusion to RIG-I Nter. In all cases, no variation in eGFP expression could be observed except for the transfection mixtures containing IHNV NV which tends to slightly increase its expression (Fig. S6A–D). As shown in Fig. 7A and B, the expression of a constitutively active form of RIG-I (RIG-I Nter) significantly activated the promoters of IFN1 and interferon-stimulated response element (ISRE) in EPC cells, whereas the co-expression of NV proteins or PPM1Bb with RIG-I Nter apparently reduced the induction of these promoters. In contrast, the co-expression of VHSV G and RIG-I Nter had no effect on the induction of both promoters, indicating a specific and negative role for NV and PPM1Bb on RIG-I signaling. We next tested whether NV and PPM1Bb proteins affect the activation of TBK1. As shown in Fig. 7C, the expression of TBK1 activated the promoter of IFN1, whereas the expression of a catalytically inactive mutant (TBK1 K38M) did not. The co-expression of NV proteins or PPM1Bb also significantly suppressed the induction of IFN1 promoter mediated by TBK1. In contrast, VHSV G protein had not effect, demonstrating a specific and negative effect of both PPM1Bb and NV on TBK1 kinase activity. Because PPM1Bb acts as a Ser/Thr protein phosphatase, we next tested whether its catalytic activity is required to antagonize TBK1 activity. As shown in Fig. 7D, the expression of a single mutant PPM1Bb-D60A or double mutant PPM1Bb-D60A + D243A, corresponding to catalytically inactive forms of PPM1B^44^,^45^, had significantly lost its capacity to inhibit RIG-I Nter induction of IFN1 promoter, suggesting that PPM1Bb enzymatic activity is important for its function. In addition, cyprinid PPM1Aa shares a similar regulatory effect on TBK1 activity as well as PPM1Aa and PPM1Bb from salmonid, indicating a conserved function of PPM1A and B proteins throughout the evolution^34^,^39^.

### Expression of VHSV NV reduces the amount of phospho-TBK1

**Figure 8:**
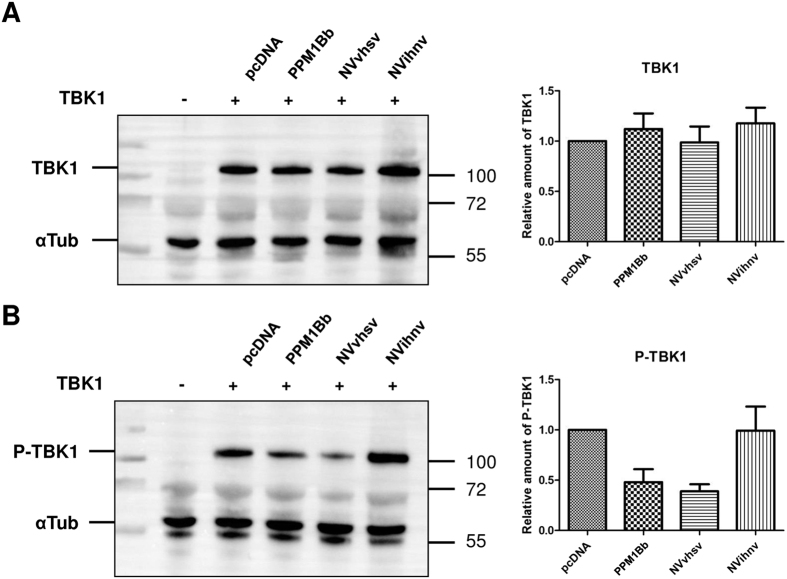
Expression of VHSV NV leads to a reduced amount of phospho-TBK1. EPC cells were transfected with 1 μg of Flag-TBK1 together with 0.5 μg of PPM1Bb or NV expression vectors. At 24 h post transfection, cell lysates were prepared and immunoblotted with either anti-Flag antibody (**A**) or anti-phospho-TBK1 (Ser172) (**B**) and anti-alpha tubulin. The relative quantification of TBK1 and Phospho-TBK1 (P-TBK1) was calculated as the ratio of the internal volumes of each TBK1 or P-TBK1 band with that of the alpha-tubulin (αTub) from the same lane measured using a Chemidoc^TM^ Touch Imaging System (BioRad). The relative amounts of TBK1 (**A**) or P-TBK1 are represented as the means of two independent experiments. Full-length blots are included in the Fig. S7.

To further explore the molecular mechanism by which NV proteins block the IFN induction, we tested whether NV proteins modulate TBK1 phosphorylation at the Ser 172 residue. As shown in Fig. 8, the overexpression of TBK1 alone leaded to its phosphorylation at the Ser 172 residue and its subsequent activation (see Fig. 7C), whereas the co-expression of PPM1Bb reduced TBK1 phosphorylation. This observation suggests that fish PPM1Bb, as their mammalian orthologs, negatively regulate the interferon response by acting as a TBK1 phosphatase. Interestingly, the expression of VHSV NV strongly reduced the amount of phospho-TBK1 indicating that VHSV NV blocks the RIG-I pathway by acting on TBK1 activation. In contrast, the amount of detected phospho-TBK1 was unchanged when co-expressed with IHNV NV, meaning that IHNV NV blocks the IFN induction by another deactivation mechanism.

## Discussion

The innate immune system acts as the first line of defense against invasion by viral pathogens. The early detection of virus infection is crucial to prevent viral replication and propagation and thus for the outcome of infection. To overcome this obstacle, viruses have evolved potent counter-defense mechanisms to evade or even inhibit key elements of host IFN responses, and multiple virus-encoded proteins are involved in those processes^31^,^32^. Among these elements, TBK1 and IKKε kinases, as critical points of convergence for many different PRR pathways that activate IRF3 and 7, are targeted by several viral proteins that modulate their activity and/or their normal interaction with their partners, including their substrates, IRF3 and IRF7^33^. While it has been previously shown that IHNV and VHSV NV proteins might be involved in the antagonism of the host innate immune and inflammatory responses, the precise mechanisms of these virus-host interactions remain fully unknown. Consistent with previous observations, infection of fish cells with NV-deletion mutant strongly induced IFN1 expression compared to wild-type and rVHSV-NVihnv chimeric virus infections, indicating that NV could perturb innate antiviral defense by blocking induction of interferon. Here, we demonstrated that both NV proteins are potent inhibitors of RIG-I-mediated IFN induction pathway. Both NV proteins strongly inhibit RIG-I- and TBK1-mediated IRF3-dependent IFN and ISRE promoter activities.

Using a reverse genetics approach to overexpress tagged-NV proteins, while retaining virus pathogenicity in rainbow trout, followed by an interactomic analysis, we identified PPM1Bb protein as a cellular partner of NV proteins. PPM1Bb is a member of the PP2C family of Ser/Thr protein phosphatases. Members of PP2C family are important physiological regulators of cell growth and of cellular stress signaling^40^. The isoenzymes PPM1A and PPM1B associated with IKK and IKK-related kinases, such as TBK1, and act as IKK phosphatases to negatively regulate antiviral, anti-apoptotic and inflammatory pathways and to maintain immune homeostasis^34^,^39^,^46^,^47^. Our study revealed a specific interaction of the NV proteins with cyprinid and salmonid PPM1Bb since the isoenzyme PPM1Aa, which is highly similar to PPM1Bb at the amino acid level (more than 73% of amino acid identity), did not interact with NV. We further showed that the expression of NV proteins has a tremendous effect on the PPM1Bb cellular relocation in close proximity to mitochondria. Mitochondria are essential for the proper induction of antiviral signaling via MAVS, the essential signaling adapter protein of the RLRs^48^. MAVS does not bind to free mitochondria but to mitochondrial associated membrane (MAM), which physically connects endoplasmic reticulum (ER) specialized domain to outer mitochondrial membrane. Upon recognition of viral RNA, RIG-I and MDA5 interact with MAVS through mutual caspase activation and recruitment domains (CARDs). This interaction results in MAVS oligomerization and the recruitment of downstream signaling factors to form MAVS signaling complexes. This leads to the recruitment of inactive TBK1 to these signaling complexes where TBK1 can be autophosphorylated due to high local concentration or phosphorylated by other kinases^49^. We showed that fish PPM1Bb interacts with fish TBK1 as found in mammalians and that TBK1 was dephosphorylated in the presence of PPM1Bb. This results in a strong inhibition of both RIG-I- and TBK1-mediated IRF3-dependent IFN and ISG promoter activities. Indeed, the overexpression of RIG-I CARD domain (RIG-I Nter) or TBK1 increases both IFN- and ISRE-promoter activities, whereas the co-expression of the wild-type PPM1Bb, but not catalytically inactive forms of PPM1Bb, significantly reduced the activity of those promoters. Collectively, our findings suggest that PPM1Bb function to maintain innate immune homeostasis in the host cells was conserved during the evolution.

Interestingly, Zixing Li and co-authors found that viral infection could increase the association between the isoenzyme PPM1A and the stimulator of interferon genes (STING), an ER-associated protein playing important functions in the activation of type I IFN in response to DNA virus infection^39^. Indeed, in cells infected by Herpes Simplex virus (HSV-1), an apparent elevation of PPM1A levels was detected in the STING immunoprecipitates at 8 hour postinfection, however the underlying mechanism by which PPM1A was recruited to this complex was not investigated. The authors demonstrated that PPM1A functions as a phosphatase to negatively regulate antiviral signaling by dephosphorylating both STING and TBK1. Consistent with this, knockdown of PPM1A led to reduced viral replication after both DNA (HSV-1) and RNA (Vesicular Stomatitis Virus) virus infections^34^,^39^. Similarly, PPM1A was recently identified as a molecular link between *Mycobacterium tuberculosis (Mtb*) infection and increased Human Immunodeficiency Virus-1 (HIV-1) susceptibility of macrophages^50^. In this study, the authors showed that increased PPM1A expression rendered macrophages highly susceptible to HIV-1 infection, while depletion of PPM1A rendered them relatively resistant to HIV-1 infection. In macrophages, both *Mtb* and HIV-1 infection induced higher level of expression of PPM1A in favor to the infection. In the present study, we did not detect any significant increase in PPM1Bb mRNA expression in fish epithelial cells at an early time point after VHSV infection (data not shown). But, at 48h postinfection, PPM1Bb was found associated with both NV proteins and relocated in close proximity to mitochondria. We further demonstrated that the expression of VHSV NV led to a strong reduction of the phosphorylated form of TBK1 corresponding to its active form. In contrast, IHNV NV expression did not reduce the amount of active TBK1 under these experimental conditions where TBK1 was overexpressed. It has to be noticed that assays to detect endogenous expression of TBK1 and P-TBK1 in EPC and other fish cell lines were unsuccessful presumably due to very low levels of TBK1 expression. Since IHNV NV also interacts with PPM1Bb, recruits it in close vicinity to mitochondria and strongly inhibits the RIG-I pathway of interferon induction, it can be speculated that IHNV NV could redirect PPM1Bb phosphatase to another substrate such as IRF3 or DDX3 which are phosphorylated by TBK1 and involved downstream it in the RLR signaling. Moreover, a strong co-localization of both PPM1Bb and IHNV NV was also observed in the nucleus where PPM1Bb could dephosphorylate another key element. Taken together, these results suggest that PPM1Bb recruitment by NV proteins is an important viral strategy for escaping RLR immune signaling. The fact that PPM1Bb was highly conserved during the evolution emphasizes the potential of these viruses to circumvent the host innate immune response in several fish species. Further studies need to focus on fine mapping of the interaction between NV proteins and PPM1Bb and whether NV could interact with PPM1Bb and PPM1Ab fish paralogs which were not tested in the present study. The putative targets of IHNV NV-PPM1Bb complexes should be investigated and PPM1Bb gene knockdown in fish cells performed in order to control whether the viruses are still able to efficiently counteract the host antiviral response. Although the available siRNA technology does not work in fish cell lines to efficiently knockdown the expression of cellular genes (for review^51^), the recent advances in CRISPR/Cas9, adapted to zebrafish and salmon, will offer the opportunity to address the importance of such recruitment for Novirhabdovirus evasion from host innate immunity^52^,^53^.

Recent studies have revealed that various cellular proteins are involved in the modulation of the RIG-I pathway such as the Asp-Glu-Ala-Asp (DEAD)-box helicase 3 (DDX3) and the elongation factor Tu GTP binding domain-containing protein 2 (EFTUD2). DDX3 belongs to the DEAD-box family of ATP-dependent RNA helicases and is localized both in the cytoplasm and the nucleus. DDX3 is a multifunctional protein involved in various RNA metabolisms, including transcription, translation, RNA splicing, RNA transport and RNA degradation. In addition, recent studies suggest that DDX3 is a component of the innate immune response against virus infections^54^. DDX3 has been shown to play roles both upstream and downstream TBK1 and IKKε kinases, stimulating the IFN production^55^,^56^. DDX3 promotes the IFN production by i) sensing viral RNA and associating with MAVS, ii) binding IKKε to facilitate IRF3 phosphorylation, and iii) being phosphorylated by TBK1 and binding the IFN-β promoter. Thus several viruses, including vaccinia virus (VACV), HIV-1 and hepatitis C virus, target DDX3 to block IFN production and even hijack it to accomplish their own replication cycles. For example, the virulence factor K7 of VACV binds DDX3 and interfere with DDX3-mediated IFN-β production^55^. On the other hand, EFTUD2 is a GTPase which is a component of the spliceosome complex which processes precursor mRNAs to produce mature mRNAs. In a recent study, the level of expression of RIG-I and MDA5 has been shown to be linked to that of EFTUD2, suggesting that EFTUD2 controls the expression of these viral sensors^38^. Moreover, silencing EFTUD2 only reduces mature mRNA of both sensors and has no effect on pre-mRNA amounts, suggesting that EFTUD2 regulates RIG-I and MDA5 through mRNA splicing. Interestingly, NV proteins were found to interact with these two cellular proteins meaning that NV proteins potentially acts on three distinct levels of the RIG-I signaling pathway by (i) recruiting PPM1Bb and dephophorylating TBK1 or another substrate involved in the pathway, (ii) interacting with EFTUD2 and interfering with the efficient maturation of RIG-I pre-mRNA, and (iii) binding DDX3 and blocking the efficient production of IFN. Further studies need to focus on the relevance and contribution of each of these specific interactions in the IFN antagonistic functions of the NV proteins.

## Methods

### Ethics Statements

All animal studies were carried out in strict accordance with the European guidelines and recommendations on animal experimentation and welfare (European Union directive 2010/63). All animal experiment procedures were approved by the local ethics committee on animal experimentation: “COMité d’ÉTHique pour l’Expérimentation Animale” #45 (COMETHEA) under permit number N°12/111. To minimize animal suffering and distress, all manipulations were carried out under light anesthesia. Anesthesia was performed by bath immersion with 0.3 mL/L of 2-phenoxy ethanol. A lethal challenge typically results in acute disease characterized by exophthalmia, anemia and punctiform hemorrhages. Therefore, fish were monitored twice a day for clinical signs and survival. In case of fish that showed typical infection symptoms, they were humanely euthanized by bath immersion using a lethal dose of 2-phenoxy ethanol (0.8 mL/L).

### Cells and virus

rVHSV were propagated in monolayer cultures of EPC cells (Epithelioma Papulosum Cyprini from fathead minnow, *Pimephales promelas*) at 15 °C as previously described^18^,^57^. Virus titers were determined by plaque assay on EPC cells under an agarose overlay (0.35% in Glasgow’s modified Eagle’s medium-HEPES 25 mM medium supplemented with 2% fetal bovine serum and 2 mM L-glutamine). At 3 to 4 days postinfection, cell monolayers were fixed with 10% formol and stained with crystal violet. Recombinant vaccinia virus expressing the T7 RNA polymerase, vTF7-3^58^, was kindly provided by B. Moss (National Institutes of Health, Bethesda, Md.). For the multiple growth kinetics, EPC cells were infected at an MOI of 0.01 PFU per cell with mutant rVHSV viruses. Supernatant aliquots (0.2 mL out of a total medium volume of 2 mL per well) were taken at different times postinfection and replaced by an equivalent volume of fresh medium. The samples were flash-frozen and analyzed later by plaque assay.

### Construction of VHSV NV-gene mutant cDNA and recombinant virus recovery

The plasmid pVHSV, which contains the complete consensus antigenomic sequence of VHSV 23–75^18^, was modified to contain an additional *SpeI* and *SnaBI* restriction site upstream and downstream the NV open reading frame (ORF), respectively (Fig. 1A). To introduce the restriction sites, the MfeI/NdeI fragment of pVHSV, containing the NV gene, was amplified by PCR using the primers 5VHSV *MfeI* and 3VHSV *NdeI* (Table S2), subcloned in pJET1.2 plasmid (CloneJET PCR cloning kit; Fermentas) and a multi-site directed mutagenesis was done using the QuikChange Multi Site-Directed Mutagenesis Kit (Strategene) and the primers NV *SpeI* MUT and NV *SnaBI* MUT (Table S2) to introduce unique enzyme restriction sites *SpeI* and *SnaBI* upstream and downstream the NV ORF, respectively. The mutated subclone was then digested with *MfeI* and *NdeI* and the resulting fragment was cloned back into the *MfeI/NdeI* window of pVHSV to create pVHSV Spe/Sna. The IHNV NV gene was amplified by PCR from pIHNV, which contains the complete consensus antigenomic sequence of IHNV 32–87^19^ by using the specific primers 5NVihn/3NVihn. The genes encoding the IHNV and VHSV NV proteins fused N-terminally to a triple flag tag (3xfNV) were obtained as following: a first PCR amplification was done to introduce a first copy of the flag tag using pIHNV or pVHSV as template and the primers 5NVihnFlagNter/3NVihn or 5NVvhsFlagNter/3NVvhs, respectively, and a second PCR amplification was then performed to introduce the two other copies of the flag tag using the products of the first PCR as template and the primers 3xFlagNV together with 3NVihn or 3NVvhs, respectively. Then the NV ORF was deleted from pVHSV Spe/Sna by *SpeI/SnaBI* digestion and replaced with NVihnv and 3xfNVvhsv, leading to pVHSV-NVihnv and pVHSV-3xfNV (G/L), respectively (Fig. 1). The deletion of the full NV gene, in the pVHSV-∆NV construct, was obtained by the removal of a 423-nucleotide fragment containing the entire NV region including the transcription signals, the NV ORF and the surrounding non-coding regions. Finally, the 3xfNV ORF from VHSV and IHNV were cloned in an expression cassette previously described^18^ and located between the N and P genes and finally transferred in the pVHSV-∆NV plasmid backbone using the unique *PsiI* enzyme restriction site, leading to pVHSV-3xfNV (N/P) plasmids (Fig. 1).

The recovery of rVHSV was carried out as described previously^18^. The recombinant virus genome structures were verified by RT-PCR amplification and nucleotide sequencing performed on viral RNA extracted from infected-cell supernatants after two passages on EPC cells.

### Experimental fish infection and virus isolation

Hundred virus-free juvenile INRA synthetic strain of rainbow trout (*Oncorhynchus mykiss*) (mean weight, 1.5 g) were infected by immersion in tanks filled with 3 liters of freshwater with mutant rVHSV viruses (final titer, 5 × 10^4 ^PFU/mL) for 2 h at 10 °C. Tanks were then filled up to 30 liters with freshwater. Controls were fish mock infected with cell culture medium under the same conditions. Mortalities were recorded every day over a period of 28 days. Virus isolation was assayed from dead fish. Bodies were homogenized in a mortar with a pestle and sea sand in Glasgow’s modified Eagle’s medium-HEPES 25 mM medium containing penicillin (200 IU/mL), streptomycin (0.2 mg/mL), kanamycin (0.2 mg/mL) and amphothericin B (2.5 mg/mL). After centrifugation at 2,000 × g for 15 min at 4 °C, the supernatant was used to inoculate EPC cells and the virus titer in each sample was determined by plaque assay.

### Co-immunoprecipitations, protein electrophoresis and Western blot assays

For the co-immunoprecipitation assays, transfected or infected cells (MOI of 1) in 6-well plates were lysed at 48 h post-transfection or infection, respectively, using 450 μL of lysis buffer (150 mM NaCl, 50 mM Tris/HCl pH = 8, 20 mM EDTA, 0.5% NP-40 and complete protease inhibitor cocktail (Roche))^59^. Aliquots of 200 μL were then incubated with 5 μg of mouse anti-Flag M2 mAb (Sigma) or rat anti-HA (Roche) and protein A-Sepharose beads (GE Healthcare) were added. After extensive washes in Tris buffer saline 1X [pH = 7.5] containing 0.5% of Tween 20, proteins were eluted from the beads with a 3X FLAG peptide (Sigma) as recommended. The samples were then separated by electrophoresis on a 4-to-12% polyacrylamide bis-Tris gel (NuPAGE Novex bis-Tris; Invitrogen) followed by Coomassie blue staining or western-blotting. After electrotransfer onto a polyvinylidene difluoride membrane (Immobilon-P; Millipore), proteins were detected with a mouse anti-Flag M2 mAb (diluted to 1/500), horseradish peroxidase (HRP)-conjugated rat anti-HA mAb (Roche; diluted to 1/1,000), rabbit anti-phospho-TBK1 (Ser172) (Cell Signaling; 1/1,000) or mouse anti-alpha tubulin (Sigma; 1/3,000). Immunodetected antigens were visualized with HRP-conjugated goat anti-mouse or anti-rabbit immunoglobulin G (PARIS; diluted to 1/5,000) by using the enhanced chemiluminescence detection system (ECL; Pierce).

### LC-MS/MS analysis and database searching and analysis

Co-immunoprecipitation products were loaded on a polyacrylamide bis-Tris gel. Proteomic analyses were performed at the PAPPSO platform (http://pappso.inra.fr), INRA, France, as described previously^60^. Protein identification was performed querying MS/MS data against protein databases (VHSV NV CBJ23833; IHNV NV Q08455; *Danio rerio*, Uniprot, 2013/11/13; *Pimephales promelas*, NCBI, 2014/02/18; *Bos Taurus*, Uniprot, 2013/13/11) together with an in house contaminant database. The final list of cellular proteins associated with NV was analyzed using the Search Tool for the Retrieval of Interacting Genes/Proteins database (STRING v10; http://string-db.org)^61^.

### Molecular cloning and sequencing of fathead minnow and salmon PPM1Aa and PPM1Bb and fathead minnow TBK1

Zebrafish (*Danio rerio*) PPM1Aa and PPM1Bb sequences (UniProt ID Q6NYP6 and Q5U386, respectively) were used as template to screen expressed sequence tags (ESTs) from trout (*Oncorhynchus mykiss*), salmon (*Salmo salar*) and fathead minnow (*Pimephales promelas*). Total RNA from fathead minnow EPC, rainbow trout RTG-2 and Atlantic salmon TO cells were extracted using RNeasy kit (QIAGEN) according to the manufacturer’s instructions. The RNA was used to generate full-length cDNAs using the SMART RACE cDNA amplification kit (BD Clontech) with universal primers provided by the manufacturer and gene-specific primers (Table S2) designed from the ESTs sequences. PCR amplifications were performed using the Advantage 2 PCR kit (BD Clontech) and following the manufacturer’s instructions. RT-PCR products were purified with QIAquick PCR purification kit (QIAGEN), cloned into the eukaryotic expression vectors pcDNA1.1/Amp (Invitrogen) and fully sequenced. The Neighbor-joining (NJ) phylogenetic tree of PPM1A and B was calculated by MEGA6 software^62^ based on a multiple alignment (using ClustalW) of full-length PPM1A and B amino-acid sequences from fish and other vertebrates and a 1000-boostrap was performed. The conserved syntenies around the PPM1A and B genes in zebrafish, mouse and human was performed based on the data from the genome assemblies available at NCBI (http://www.ncbi.nlm.nih.gov/) and using Genomicus PhyloView of Genomicus v86.01^63^. The specific primers used to amplify by RT-PCR the TBK1 gene expressed by EPC cells were designed from fathead minnow EST sequences found in GenBank using the goldfish (*Carassius auratus*) sequence of TBK1 gene (GenBank JF970228)^64^.

### Transfection, fluorescence microscopy and luciferase activity assay

EPC cells were plated into 6-well plates at a concentration of 5 × 10^6^ cells per well 24 h prior to transfection by electroporation (Amaxa Biosystems; Lonza). Cells were trypsinized and resuspended in 100 μL of solution T. Cells were then mixed with 0.5 to 4 μg of plasmid DNA and electroporated using the program T-020. At 48 h post-transfection, cells were fixed or lysed for further experiments.

For immunofluorescence microscopy, the mitochondria were stained using 500 nM of MitoTracker DeepRed FM (Invitrogen). Cell monolayers were then fixed with a mixture of alcohol and acetone [1:1 (v/v)] or with 80% methanol (after mitochondria staining) at −20 °C for 15 min. Antigen detection was performed by incubation with mouse anti-Flag M2 (1/1,000) or rabbit anti-HA mAbs (1/50; Sigma) diluted in PBS1x containing 0.05% of Tween 20 for 45 min at room temperature. Cells were then washed three times, incubated with Alexa Fluor 488-conjugated anti-mouse and Alexa Fluor 594-conjugated anti-rabbit immunoglobulins (Invitrogen) for 45 min at room temperature and washed again. Cell monolayers were then visualized directly with a UV-light microscope (Carl Zeiss) after mounting of the coverslips using Pro-Long Gold antifade reagent with 4′, 6-diamidino-2-phenylindole (DAPI) medium (Invitrogen).

For IFN promoter reporter assays, EPC cells (5 × 10^6^ cells per well of 6-well plate) transfected (see above) with pIFNpro-LUC^65^ or pISRE-LUC (Clontech) together with various plasmid DNA constructs. At 24 h post transfection, cell lysates were performed using 400 μL of cell culture lysis reagent per well according to the manufacturer’s instructions (luciferase reporter assay system - Promega). eGFP expression from peGFP or pRIG-I Nter-eGFP^66^ was measured from 100 μL of cell lysates on a Tecan infinite M200 Pro reader using an excitation wavelength of 480 nm and an emission wavelength of 510 nm. Luciferase activity was then measured by adding 100 μL of luciferase assay reagent. Values of luciferase activities were normalized to the levels of eGFP fluorescence. The fold-induction was calculated as the ratio of stimulated versus unstimulated (pcDNA alone) samples. All data shown are representatives of at least three independent experiments.

## Additional Information

**How to cite this article**: Biacchesi, S. *et al*. NV Proteins of Fish Novirhabdovirus Recruit Cellular PPM1Bb Protein Phosphatase and Antagonize RIG-I-Mediated IFN Induction. *Sci. Rep.* **7**, 44025; doi: 10.1038/srep44025 (2017).

**Publisher's note:** Springer Nature remains neutral with regard to jurisdictional claims in published maps and institutional affiliations.

## Supplementary Material

Supplementary Information
