# Supplementary material for: NV Proteins of Fish Novirhabdovirus Recruit Cellular PPM1Bb Protein Phosphatase and Antagonize RIG-I-Mediated IFN Induction
Source: Sci Rep. 2017 Mar 9;7:44025. doi: 10.1038/srep44025 (PMC5343655; doi:10.1038/srep44025)

# NV Proteins of Fish Novirhabdovirus Recruit Cellular PPM1Bb Protein Phosphatase and Antagonize RIG-I-Mediated IFN Induction.

**Supplementary Information**

Stéphane Biacchesi*^1^, Emilie Mérour^1^, Didier Chevret^2^, Annie Lamoureux^1^, Julie Bernard^1^, Michel Brémont^1^.

^1^ VIM, INRA, Université Paris-Saclay, 78350, Jouy-en-Josas, France.

^2^ PAPPSO, Micalis Institute, INRA, AgroParisTech, Université Paris-Saclay, 78350 Jouy-en-Josas, France.

Running title: Recruitment of PPM1Bb by NV proteins

Key words: Novirhabdovirus; NV; PPM1Bb; RIG-I; Interferon.

*Corresponding author:

Phone: (33) 1 34 65 26 38

Fax: (33) 1 34 65 26 21

e-mail: [**stephane.biacchesi@jouy.inra.fr**](mailto:ubuchholz@niaid.nih.gov)

# Table S1: List of cellular and viral proteins associated with 3xFlag-tagged NV.

**A Viral Proteins:**

| Gene Bank # | Gene | PROTEIN | Sequence Coverage^a^ | Unique Peptides^b^ | MW (kDa) |
| --- | --- | --- | --- | --- | --- |
| CBJ23833 vhsv  Q08455 ihnv | NV | non virion protein | 84/54 | 21/8 | 16 |
| CBJ23834 | L | large viral polymerase | 41/16 | 60/15 | 224 |
| CBJ23829 | N | nucleoprotein | 71/40 | 26/13 | 44 |
| CBJ23831 | M | matrix protein | 84/54 | 19/6 | 22 |
| CBJ23832 | G | glycoprotein | 38/7 | 11/3 | 57 |
| CBJ23830 | P | phosphoprotein | 39/_ | 7/_ | 24 |

**a.** Percentage of sequence coverage obtained for NV and the other detected viral proteins (IHNV/VHSV).

**b.** Number of different peptides that allowed identification of NV and other viral proteins (IHNV/VHSV).

**B Cellular Proteins:**

1. **Both IHNV and VHSV NV proteins:**

This list is restricted to cellular proteins detected at least once for both NV proteins and in both analyses. Grey highlights indicate the proteins for which a direct interaction with NV was validated.

| Gene ID  *Homo sapiens* | UniProt ID  *Danio rerio* | GENE | PROTEIN | Sequence Coverage^a^ | Unique Peptides^b^ | MW (kDa) |
| --- | --- | --- | --- | --- | --- | --- |
| 10985 | F1R8B4 | GCN1L1 | general control of amino-acid synthesis 1-like 1 | 15/4 | 26/6 | 292 |
| 3312 | Q6NYR4 | HSPA8 | heat shock 70kDa protein 8 | 24/17 | 10/7 | 71 |
| 818 | Q4V9P8 | CAMK2G | calcium/calmodulin-dependent protein kinase II gamma | 19/8 | 9/2 | 62 |
| 816 | A2BGW3 | CAMK2B | calcium/calmodulin-dependent protein kinase II beta | 20/6 | 9/2 | 68 |
| 10294 | Q7ZUP5 | DNAJA2 | DnaJ (Hsp40) homolog, subfamily A, member 2 | 15/11 | 7/3 | 45 |
| 81855 | B8JJ32 | SFXN3 | sideroflexin 3 | 30/20 | 6/5 | 35 |
| 8604 | A0A0R4IHT0 | SLC25A12 | solute carrier family 25 (aspartate/glutamate carrier), member 12 | 8/5 | 5/2 | 75 |
| 23530 | Q6NYQ7 | NNT | nicotinamide nucleotide transhydrogenase | 6/2 | 5/2 | 113 |
| 23352 | F1R984 | UBR4 | ubiquitin protein ligase E3 component n-recognin 4 | 8/1 | 25/3 | 570 |
| 476 | Q9DGL6 | ATP1A1 | ATPase, Na+/K+ transporting, alpha 1 polypeptide | 18/1 | 12/1 | 113 |
| 55832 | Q5TYZ5 | CAND1 | cullin-associated and neddylation-dissociated 1 | 14/1 | 11/1 | 136 |
| 10075 | E7F8Q5 | HUWE1 | HECT, UBA and WWE domain containing 1, E3 ubiquitin protein ligase | 3/1 | 11/1 | 498 |
| 5901 | P79735 | RAN | RAN (ras-related nuclear protein), member RAS oncogene family | 43/46 | 10/10 | 24 |
| 10059 | Q7SXN5 | DNM1L | dynamin 1-like | 20/1 | 8/1 | 77 |
| 163 | Q6NYJ9 | AP2B1 | adaptor-related protein complex 2, beta 1 subunit | 10/6 | 7/4 | 105 |
| 9343 | F1Q6N0 | EFTUD2 | elongation factor Tu GTP binding domain containing 2 | 7/9 | 5/7 | 109 |
| 31 | F6P055 | ACACA | acetyl-CoA carboxylase alpha | 3/1 | 6/1 | 266 |
| 1654 | B0S6P6 | DDX3X | DEAD (Asp-Glu-Ala-Asp) box helicase 3 | 9/8 | 5/5 | 78 |
| 3329 | Q803B0 | HSPD1 | heat shock 60kDa protein 1 (chaperonin) | 11/10 | 5/4 | 61 |
| 824 | E7FBY5 | CAPN2 | calpain 2, (m/II) large subunit | 9/6 | 5/4 | 78 |
| 1785 | A0A0R4IIR1 | DNM2 | dynamin 2 | 7/2 | 5/3 | 86 |
| 160 | F1QZ45 | AP2A1 | adaptor-related protein complex 2, alpha 1 subunit | 7/2 | 5/2 | 104 |
| 30846 | Q6P3J7 | EHD2 | EH-domain containing 2 | 11/5 | 5/2 | 61 |
| 10061 | F1QZ64 | ABCF2 | ATP-binding cassette, sub-family F (GCN20), member 2 | 9/8 | 4/4 | 70 |
| _ | F1R8L3 | RPZ5 | rapunzel 5 [ Danio rerio (zebrafish) ] | 12/5 | 4/2 | 45 |
| 3032 | F1QYX8 | HADHB | hydroxyacyl-CoA dehydrogenase/3-ketoacyl-CoA thiolase/enoyl-CoA hydratase (trifunctional protein), beta subunit | 10/1 | 4/1 | 50 |
| 56926 | Q6NZ07 | NCL1 | Nicalin-1 (NCLN) | 7/5 | 3/2 | 64 |
| 3608 | Q6NZ06 | ILF2 | Interleukin enhancer-binding factor 2 | 9/1 | 3/1 | 43 |
| 26121 | Q7SXM7 | PRPF31 | pre-mRNA processing factor 31 | 6/4 | 2/2 | 56 |
| 6836 | Q6DGJ8 | SURF4 | surfeit gene 4 | 11/10 | 2/2 | 30 |
| 1717 | Q7SXF1 | DHCR7 | 7-dehydrocholesterol reductase | 5/4 | 2/2 | 55 |
| 58472 | F1QYT2 | SQRDL | sulfide quinone reductase-like | 6/6 | 2/2 | 53 |
| 3182 | F8W4E3 | HNRNPAB | heterogeneous nuclear ribonucleoprotein A/B | 6/5 | 2/2 | 40 |
| 5495 | Q5U386 | PPM1Bb | protein phosphatase, Mg2+/Mn2+ dependent, 1Bb | 1/6 | 1/2 | 42 |
| 64210 | E7FBU4 | MMS19 | MMS19 homolog, cytosolic iron-sulfur assembly component | 2/1 | 2/1 | 113 |

**a.** Highest percentage of sequence coverage obtained for each cellular protein associated to NV (IHNV/VHSV).

**b.** Highest number of different peptides that allowed identification of each cellular protein associated to NV (IHNV/VHSV).

1. **IHNV NV protein:**

This list is restricted to cellular proteins detected in both analyses.

| Gene ID  *Homo sapiens* | UniProt ID  *Danio rerio* | GENE | PROTEIN | Sequence Coverage^a^ | Unique Peptides^b^ | MW (kDa) |
| --- | --- | --- | --- | --- | --- | --- |
| 5214 | E7FDP2  E9QFJ1 | PFKP | phosphofructokinase | 10 | 5 | 86 |
| 3843 | B8JHR9 | IPO5 | importin 5 (KPNB3, RANBP5) | 8 | 5 | 123 |
| 23244 | A1L1F4 | PDS5A | PDS5 cohesin associated factor A | 4 | 4 | 149 |
| 1650 | Q6NYS8 | DDOST | dolichyl-diphosphooligosaccharide-protein glycosyltransferase non-catalytic subunit | 10 | 4 | 51 |
| 2182 | F1R452 | ACSL4 | acyl-CoA synthetase long-chain family member 4 | 5 | 3 | 79 |
| 3837 | A0A0R4IMZ8 | KPNB1 | karyopherin subunit beta 1 (importin IPO1) | 9 | 3 | 97 |
| 4174 | Q7ZVL6 | MCM5 | minichromosome maintenance complex component 5 | 3 | 2 |  |
| 8672 | E7EYD8 | EIF4G3 | eukaryotic translation initiation factor 4 gamma 3 | 2 | 2 | 176 |
| 1718 | F1QUM4 | DHCR24 | 24-dehydrocholesterol reductase | 5 | 2 | 60 |
| 387 | Q6DHE8 | RHOA | ras homolog family member A | 17 | 2 | 22 |
| 9690 | Q1LY19 | UBE3C | ubiquitin protein ligase E3C | 2 | 2 | 124 |

**a.** Highest percentage of sequence coverage obtained for each cellular protein associated to IHNV NV.

**b.** Highest number of different peptides that allowed identification of each cellular protein associated to IHNV NV.

1. **VHSV NV protein:**

This list is restricted to cellular proteins detected in both analyses.

| Gene ID  *Homo sapiens* | UniProt ID  *Danio rerio* | GENE | PROTEIN | Sequence Coverage^a^ | Unique Peptides^b^ | MW (kDa) |
| --- | --- | --- | --- | --- | --- | --- |
| 168 | F1QGW6 | EIF2S3 | eukaryotic translation initiation factor 2 subunit gamma | 9 | 2 | 51 |

**a.** Highest percentage of sequence coverage obtained for the cellular protein associated to VHSV NV.

**b.** Highest number of different peptides that allowed identification of the cellular protein associated to VHSV NV.

# Table S2: Primers used to construct VHSV NV-gene mutant cDNA.

| **Primer** | **Sequence (5’ to 3’)^a^** | **Location^b^** | **Restriction site** |
| --- | --- | --- | --- |
| **5VHSV MfeI** | CAGGGTGGTCAAGGCAATTGTGGCAGGC | 3661-3688 | MfeI |
| **3VHSV NdeI** | GTACTCCACATATGTTACTGCGCCAAGC | 5644-5617 | NdeI |
| **NV SpeI MUT** | AAATGGCACCTTTATGATAAA**CT**A**GT**ATGGCGACCCAACCCGCGC | 4538-4582 | SpeI |
| **NV SnaBI MUT** | GGCTCTGGGCTCACCTCCTGA**TA**C**GTA**CCGCCGTCTCTCAGATAG | 4912-4956 | SnaBI |
| **5NVihn** | GGGACTAGTATGGACCACCGCGACATAAAC |  | SpeI |
| **3NVihn** | GGGTACGTACTATCTGGGATAAGCAAG |  | SnaBI |
| **5NVvhsFlagNter** | GGGACTAGT*ATGGATTACAAGGATGACGATGACAAGGTG*GCGACCCAACCCGCGCTC |  | SpeI |
| **3NVvhs** | GGGTACGTATCAGGAGGTGAGCCCAGAGCCTC |  | SnaBI |
| **5NVihnFlagNter** | GGGACTAGT*ATGGATTACAAGGATGACGATGACAAG*GACCACCGCGACATAAACACG |  | SpeI |
| **3xFlagNV** | GGGACTAGT*ATGGACTACAAAGACCATGACGGTGATTATAAAGATCATGACATCGATTACAAGGATGACGATGACAAGG* |  | SpeI |
| **5PPM1Aa_EPC_Eco** | GGGGAATTCATGGGTGCATTTCTCGATAAGCC |  | EcoRI |
| **3PPM1Aa_EPC_Xho** | GGGCTCGAGCTACCACATGTCATCCGTGGATGC |  | XhoI |
| **5PPM1Bb_EPC_Eco** | GGGGAATTCATGGGGGCCTTCTTGGACAAACCG |  | EcoRI |
| **3PPM1Bb_EPC_Xho** | GGGCTCGAGCTACCAGGGGTCGTCAAGGTCTCCTCC |  | XhoI |
| **PPM1Bb_D60A** | CTGGTCCTTCTTCGCGGTGTACG**C**CGG**C**CACGCTGGCTCCCGCG |  | NaeI |
| **PPM1Bb_D243A** | GATGAATTTGTGGTGCTCGCCTGCG**CC**GG**C**GTCTGGGATGTGATGAC |  | NaeI |
| **5PPM1Aa_Sal_Eco** | CCCGAATTCATGGGGGCGTTTTTGGACAAGCC |  | EcoRI |
| **3PPM1Aa_Sal_Xho** | GGGCTCGAGTTACCACATGTCGTCTGTCGATGC |  | XhoI |
| **5PPM1Bb_Sal_Eco** | CCCGAATTCATGGGGGCGTTCCTGGACAAGCCTAAG |  | EcoRI |
| **3PPM1Bb_Sal_Xho** | GGGCTCGAGCTACCAGTGGTCCTCCAGATCACACC |  | XhoI |
| **5TBK1_EPC_Eco** | GGGGAATTCATGGCGGAAAATATGTCTCTACTTGGTCTCG |  | EcoRI |
| **3TBK1_EPC_Xho** | GGGCTCGAGTCAGTTGCTAAGCTTGCACAGAGACACAG |  | XhoI |
| **TBK1_K38M** | GGGCGATCTGTACGCGGTCA**TG**GTGTTTAATAACCTGAGCTTTC |  |  |
| **5EPC_EFTUD2_Eco** | CCCGAATTCATGGAGACGGATCTTTATGATGAGTTCGG |  | EcoRI |
| **3EPC_EFTUD2_Sal** | CCCGTCGACTCACATCGGGTAGTTGAGCACCACATCTTGTTTGG |  | SalI |
| **5EPC_DDX3_Eco** | CCCGAATTCATGAGTCATGTGGCCGTCGAG |  | EcoRI |
| **3EPC_DDX3_Xho** | GGGCTCGAGTCAGTTACCCCACCAGTCCACCTG |  | XhoI |
| **5EPC_TRIM25_Eco** | CCCGAATTCATGGCGGAAAATATGTCTCTACTTGGTCTCG |  | EcoRI |
| **3EPC_TRIM25_Xho** | GGGCTCGAGTCAGTTGCTAAGCTTGCACAGAGACACAG |  | XhoI |
| **5EPC_SFXN3_Bgl** | CCCAGATCTATGTCTGGAGATTTGCCCTTGAATATCAAC |  | BglII |
| **3EPC_SFXN3_Xho** | GGGCTCGAGCTACAGACCCTTGTTGAAAAAAACAGTG |  | XhoI |

**a.** Restriction enzyme sites are underlined; mutated nucleotides are boldfaced; Flag tag sequences are italicized.

**b.** Numbers refer to the VHSV 23-75 nucleotide sequence (GenBank accession no. FN665788).

# Table S3: Protein accession numbers.

|  | Accession Number/Protein_ID | Length (aa) |
| --- | --- | --- |
| EPC PPM1Aa* | LT174675/CZQ42526.1 | 390 |
| EPC PPM1Bb* | LT174674/CZQ42525.1 | 382 |
| Zebra PPM1Aa | XP_005156831 | 384 |
| Zebra PPM1Ab | AAI25895 | 333 |
| Zebra PPM1Ba | NP_571473 | 390 |
| Zebra PPM1Bb | NP_001007314 | 382 |
| Pike PPM1Aa | XP_010877131 | 384 |
| Pike PPM1Ab | XP_010880701 | 340 |
| Pike PPM1Ba | XP_010887075 | 386 |
| Pike PPM1Bb | XP_010862679 | 380 |
| Trout PPM1Aa* | LT174680/CZQ42531.1 | 382 |
| Trout PPM1Ab | CDQ71870 | 342 |
| Trout PPM1Ba | Cdq66274 | 386 |
| Trout PPM1Bb* | LT174681/CZQ42532.1 | 380 |
| Salmon PPM1Aa* | LT174682/CZQ42533.1 | 384 |
| Salmon PPM1Ab | XP_014000151 | 342 |
| Salmon PPM1Ba | XP_014066066 | 386 |
| Salmon PPM1Bb* | LT174683/CZQ42534.1 | 380 |
| Human PPM1A | NP_066283 | 382 |
| Human PPM1B | NP_002697 | 479 |
| Mouse PPM1A | NP_032936 | 382 |
| Mouse PPM1B | NP_001152968 | 477 |
| EPC SFXN3* | LT174677/CZQ42528.1 | 322 |
| EPC EFTUD2* | LT174678/CZQ42529.1 | 971 |
| EPC DDX3* | LT174679/CZQ42530.1 | 707 |
| EPC TRIM25* | LT174676/CZQ42527.1 | 662 |
| EPC TBK1* | LT174673/CZQ42524.1 | 727 |

*Amplified and fully sequenced in this study. a or b identify both paralogs of each fish species.

# Table S4: Deduced amino acid sequence identity and similarity of fish and mammalian PPM1A and PPM1B molecules.

1. **Percentage of aa identity/similarity between PPM1A** orthologs:

|  | Zebra Aa | Pike Aa | Salmon Aa | Trout Aa | Human A | Mouse A | Pike Ab | Salmon Ab | Trout Ab | Zebra Ab |
| --- | --- | --- | --- | --- | --- | --- | --- | --- | --- | --- |
| Carp Aa | 96/98 | 90/96 | 88/94 | 90/96 | 85/95 | 86/95 | 86/93 | 87/93 | 87/93 | 86/93 |
| Zebra Aa |  | 90/95 | 88/93 | 90/95 | 85/94 | 85/94 | 87/93 | 87/93 | 88/93 | 87/93 |
| Pike Aa |  |  | 94/97 | 95/97 | 86/93 | 86/93 | 85/91 | 86/92 | 86/92 | 87/92 |
| Salmon Aa |  |  |  | 95/98 | 84/92 | 84/92 | 84/90 | 84/92 | 85/92 | 84/90 |
| Trout Aa |  |  |  |  | 85/93 | 86/94 | 86/91 | 86/92 | 87/92 | 86/91 |
| Human A |  |  |  |  |  | 98/99 | 82/91 | 82/91 | 82/91 | 84/92 |
| Mouse A |  |  |  |  |  |  | 83/91 | 83/92 | 82/92 | 84/92 |
| Pike Ab |  |  |  |  |  |  |  | 94/96 | 94/96 | 90/93 |
| Salmon Ab |  |  |  |  |  |  |  |  | 99/99 | 90/92 |
| Trout Ab |  |  |  |  |  |  |  |  |  | 91/93 |

1. **Percentage of aa identity/conserved between PPM1B** orthologs:

|  | Zebra Bb | Human B | Mouse B | Pike Ba | Salmon Ba | Trout Ba | Zebra Ba | Pike Bb | Trout Bb | Salmon Bb |
| --- | --- | --- | --- | --- | --- | --- | --- | --- | --- | --- |
| Carp Bb | 96/99 | 84/93 | 85/92 | 84/90 | 83/89 | 82/89 | 81/91 | 81/90 | 81/90 | 80/89 |
| Zebra Bb |  | 85/93 | 84/92 | 85/90 | 83/89 | 83/89 | 81/90 | 82/90 | 82/91 | 81/90 |
| Human B |  |  | 94/98 | 82/89 | 81/88 | 81/88 | 77/89 | 80/89 | 80/89 | 78/88 |
| Mouse B |  |  |  | 82/89 | 81/87 | 80/88 | 77/88 | 80/89 | 80/89 | 79/88 |
| Pike Ba |  |  |  |  | 94/96 | 93/96 | 82/91 | 82/89 | 81/89 | 80/88 |
| Salmon Ba |  |  |  |  |  | 94/96 | 81/89 | 79/88 | 79/87 | 78/87 |
| Trout Ba |  |  |  |  |  |  | 80/89 | 79/87 | 79/87 | 77/86 |
| Zebra Ba |  |  |  |  |  |  |  | 78/87 | 78/86 | 77/86 |
| Pike Bb |  |  |  |  |  |  |  |  | 98/99 | 96/97 |
| Trout Bb |  |  |  |  |  |  |  |  |  | 96/98 |

1. **Percentage of aa identity/similarity between PPM1A(a) and PPM1B(b) in selected species**:

| EPC | 75/86 |
| --- | --- |
| Zebra | 75/87 |
| Salmon | 73/84 |
| Trout | 73/86 |
| Pike | 74/86 |
| Human | 76/88 |
| Mouse | 76/89 |

#
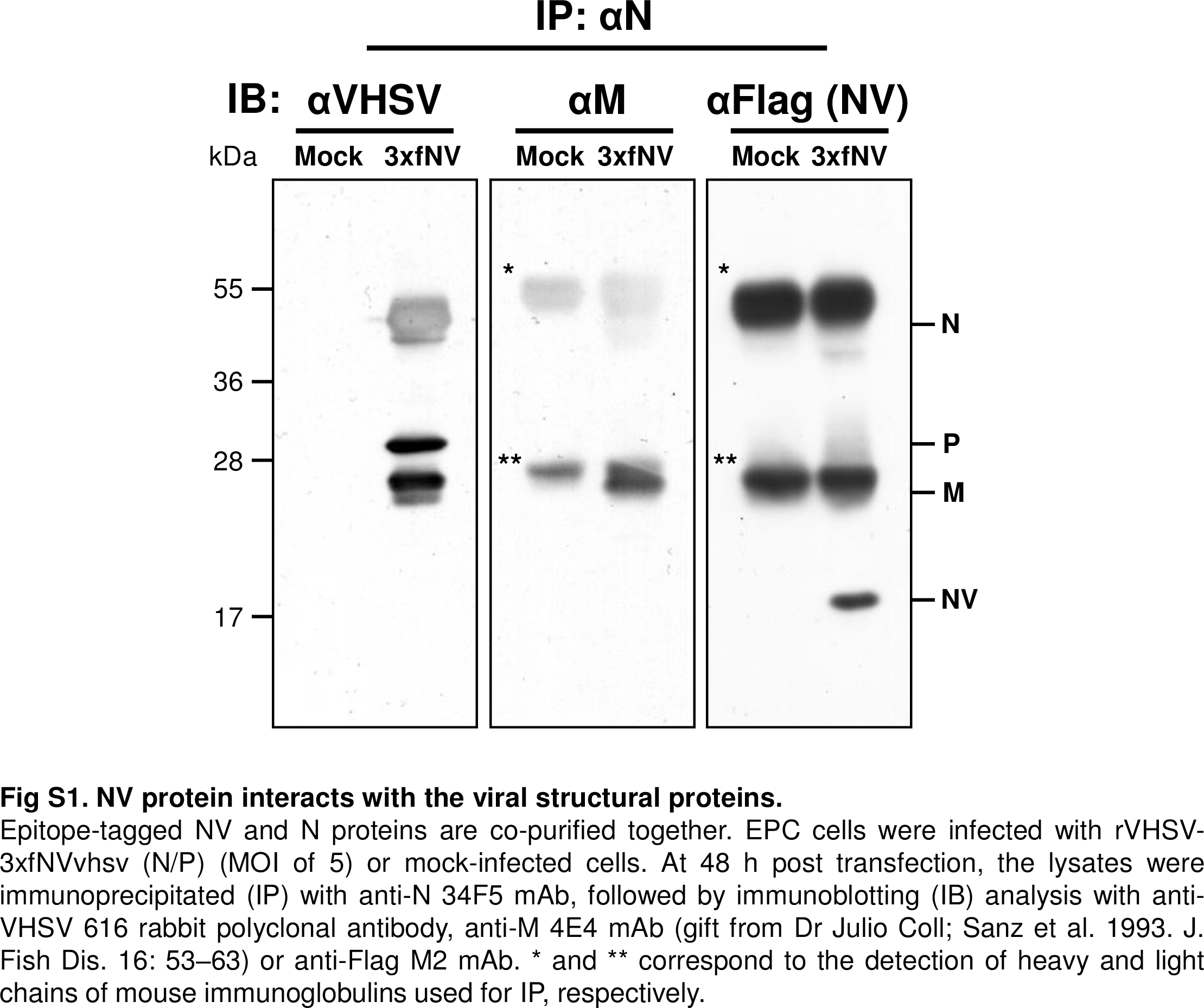


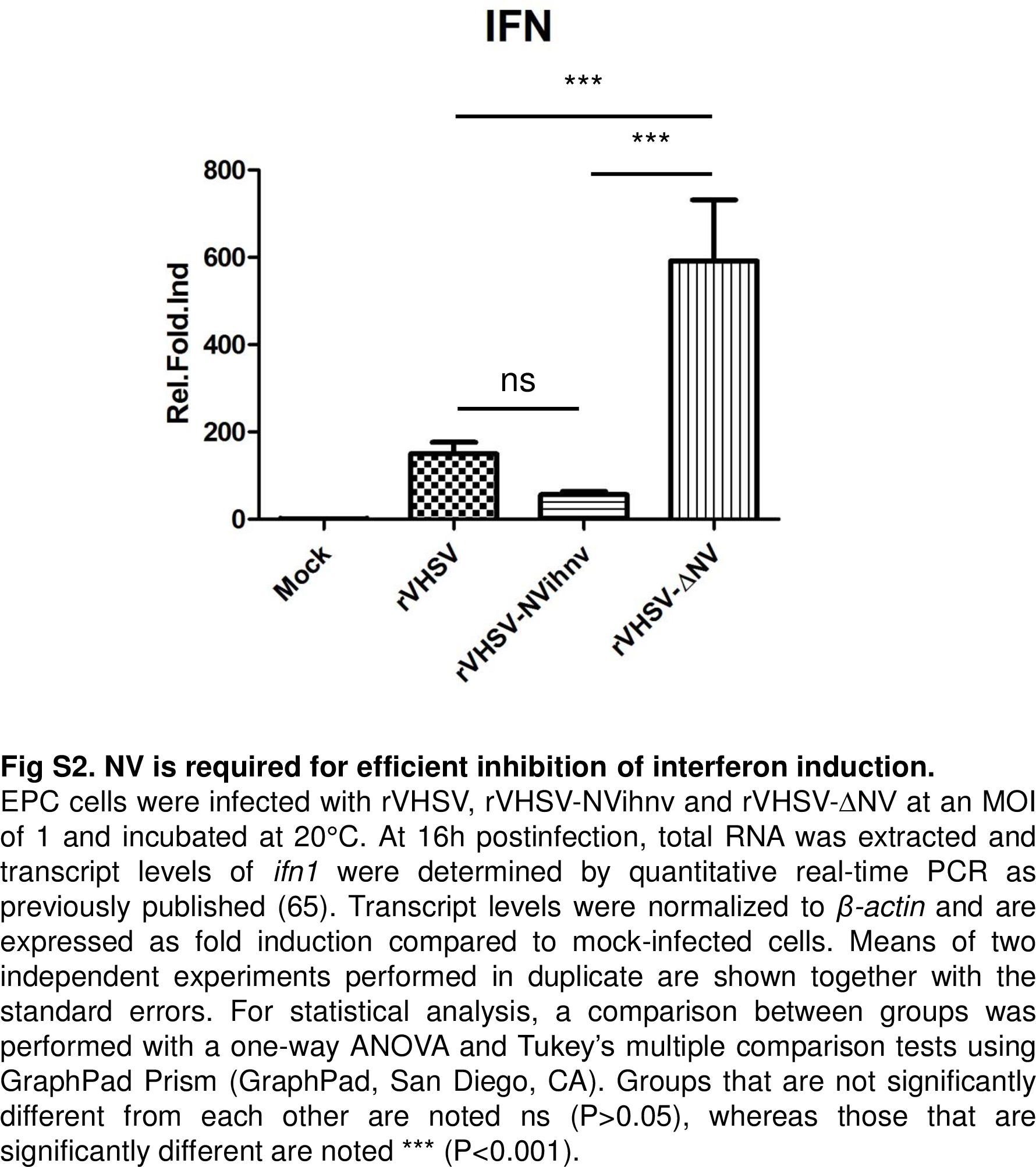


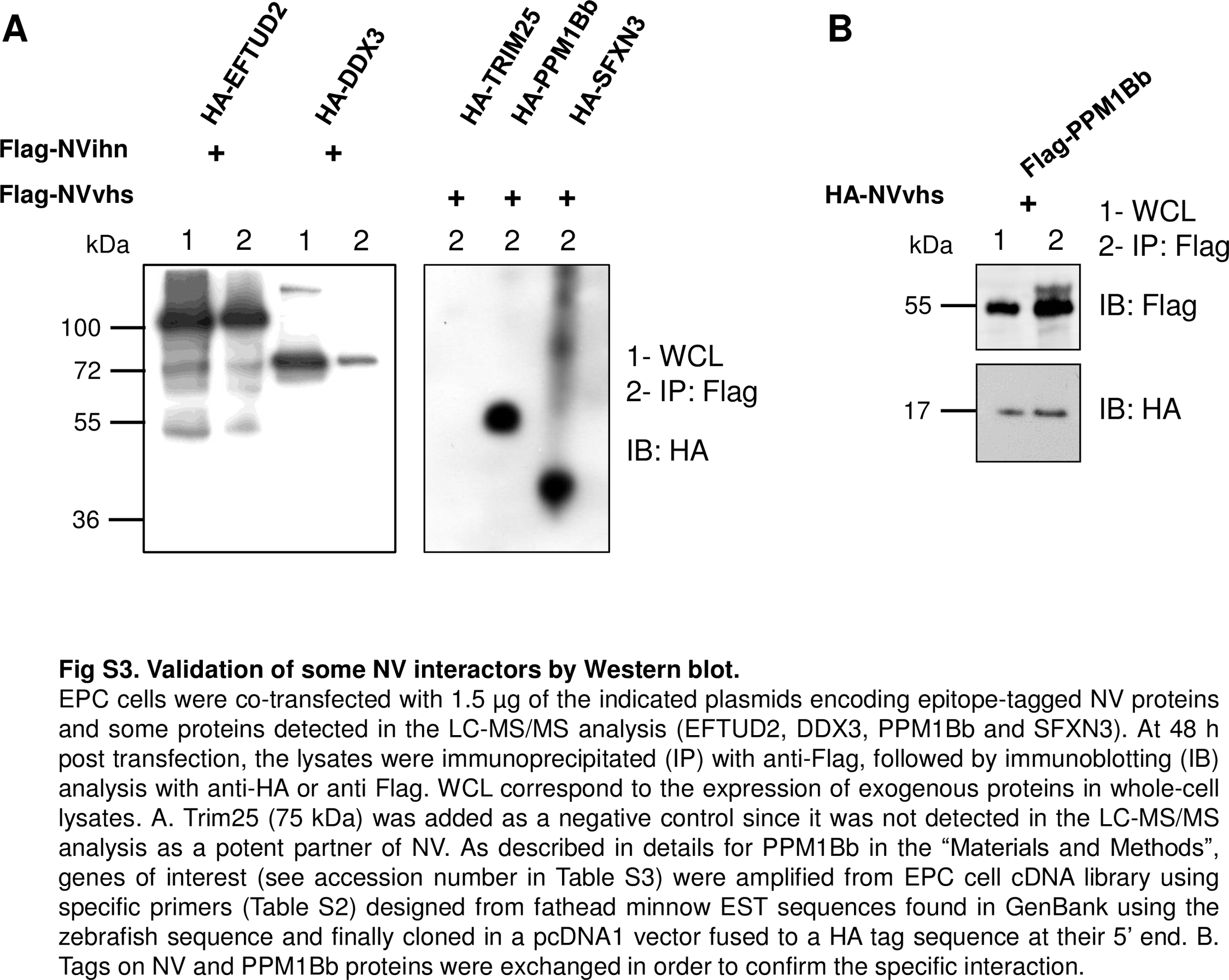


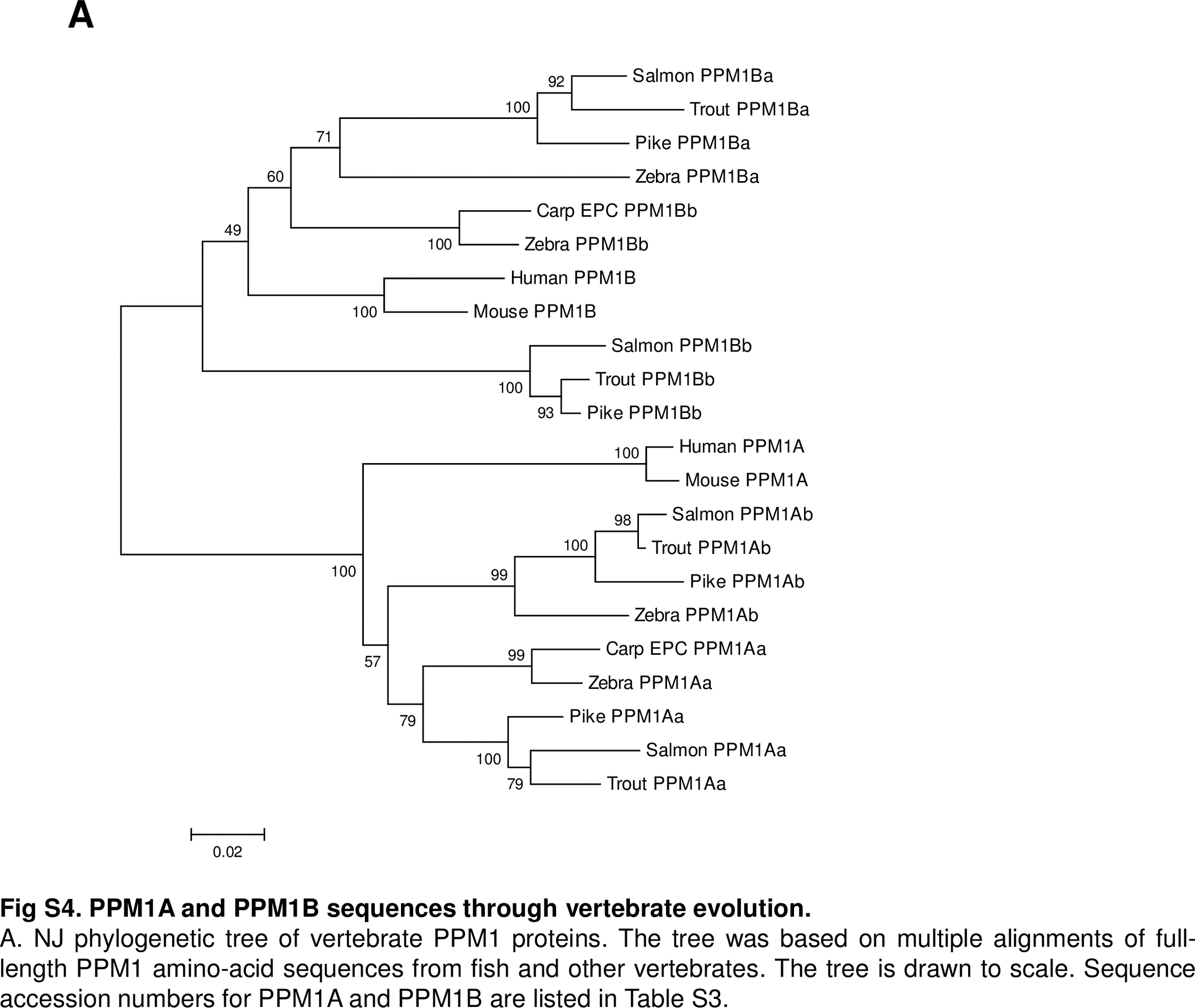


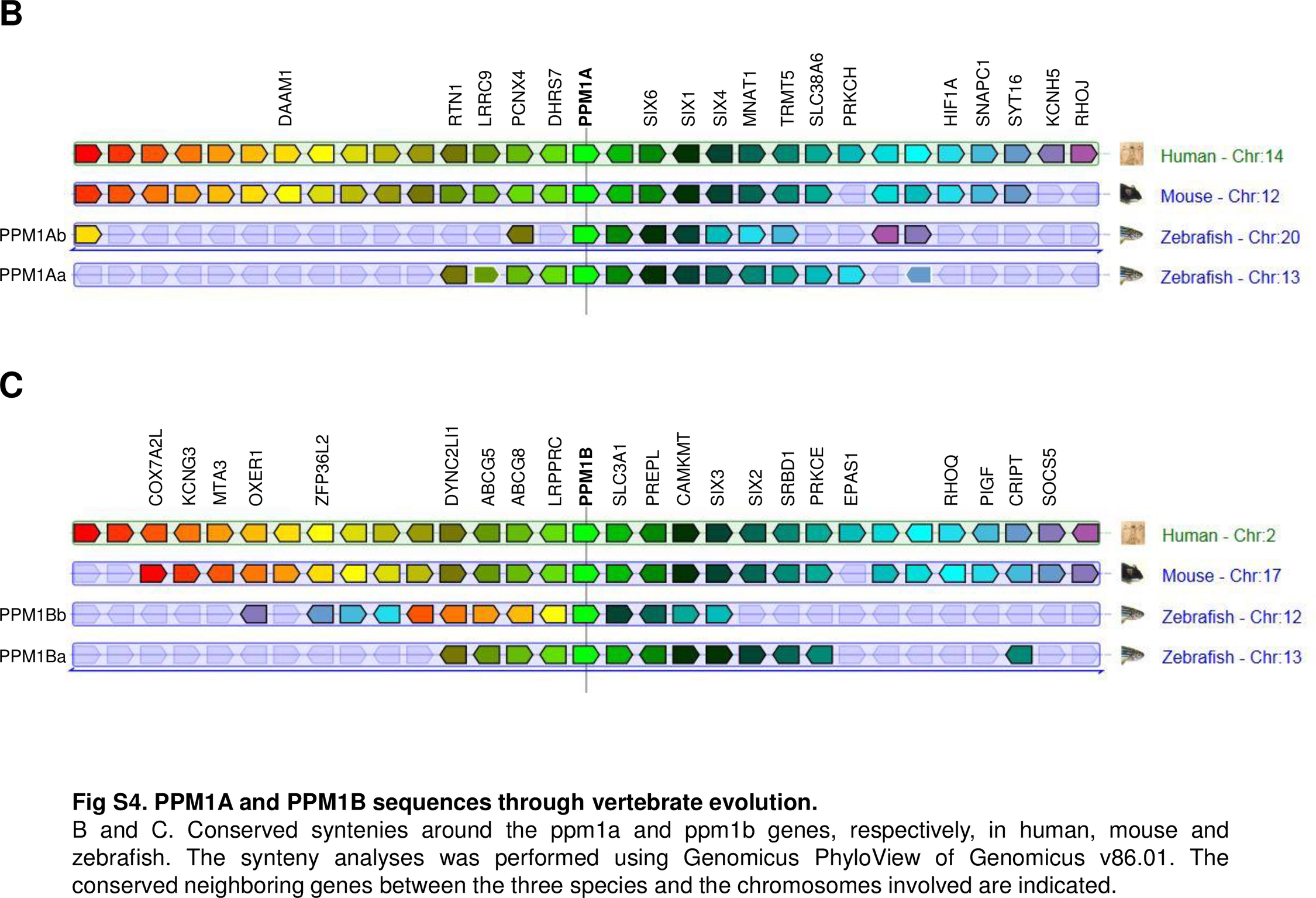


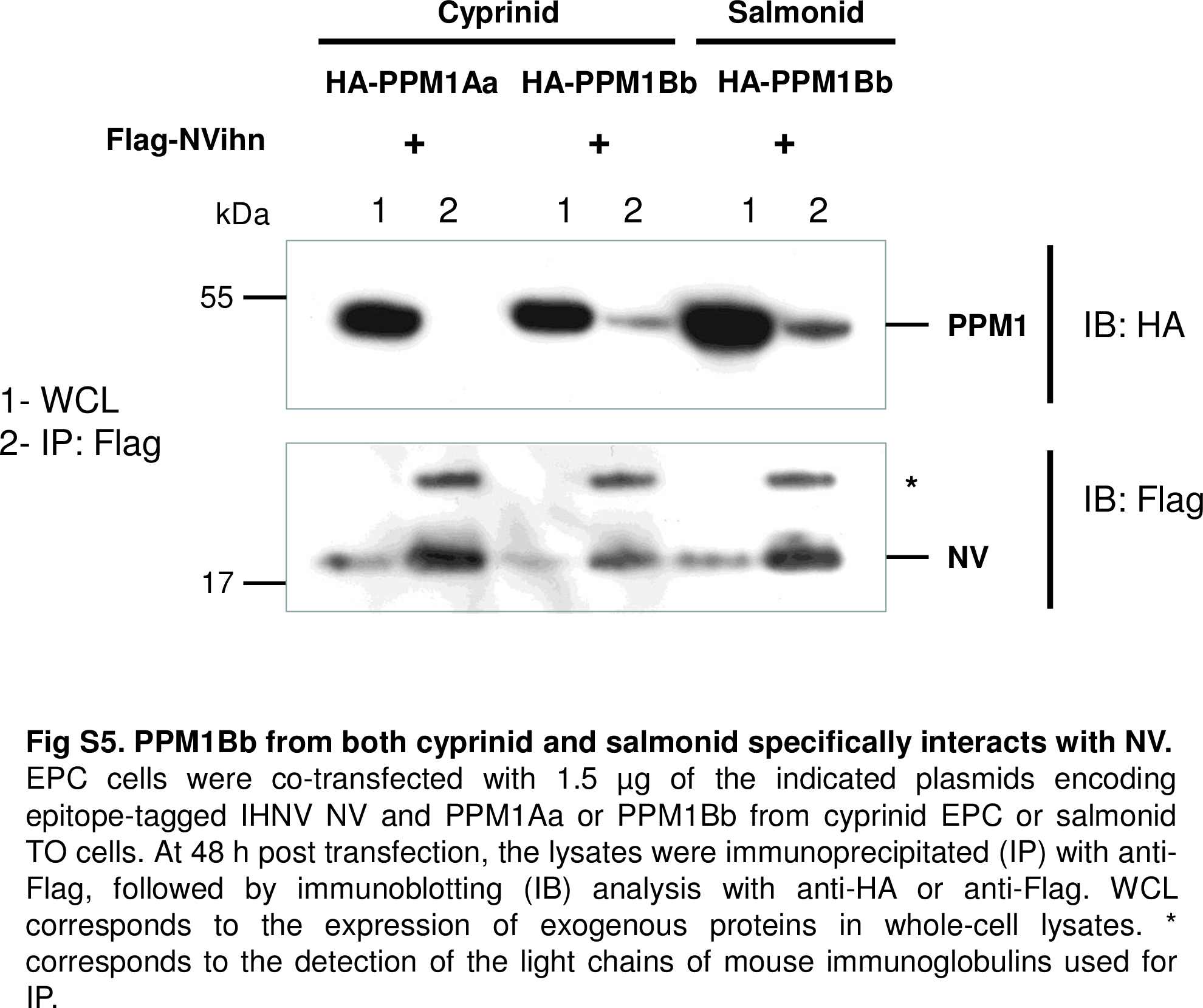


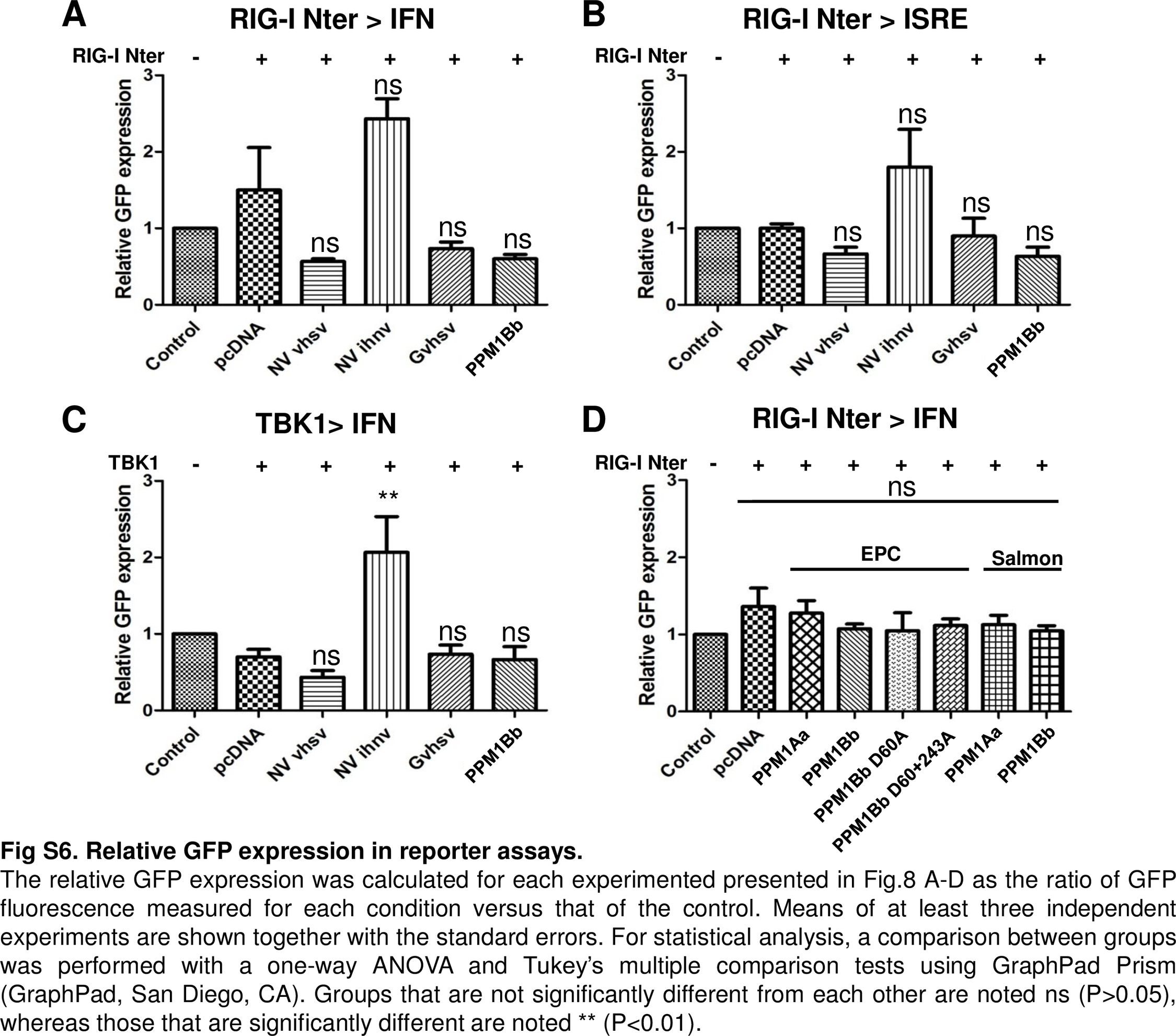


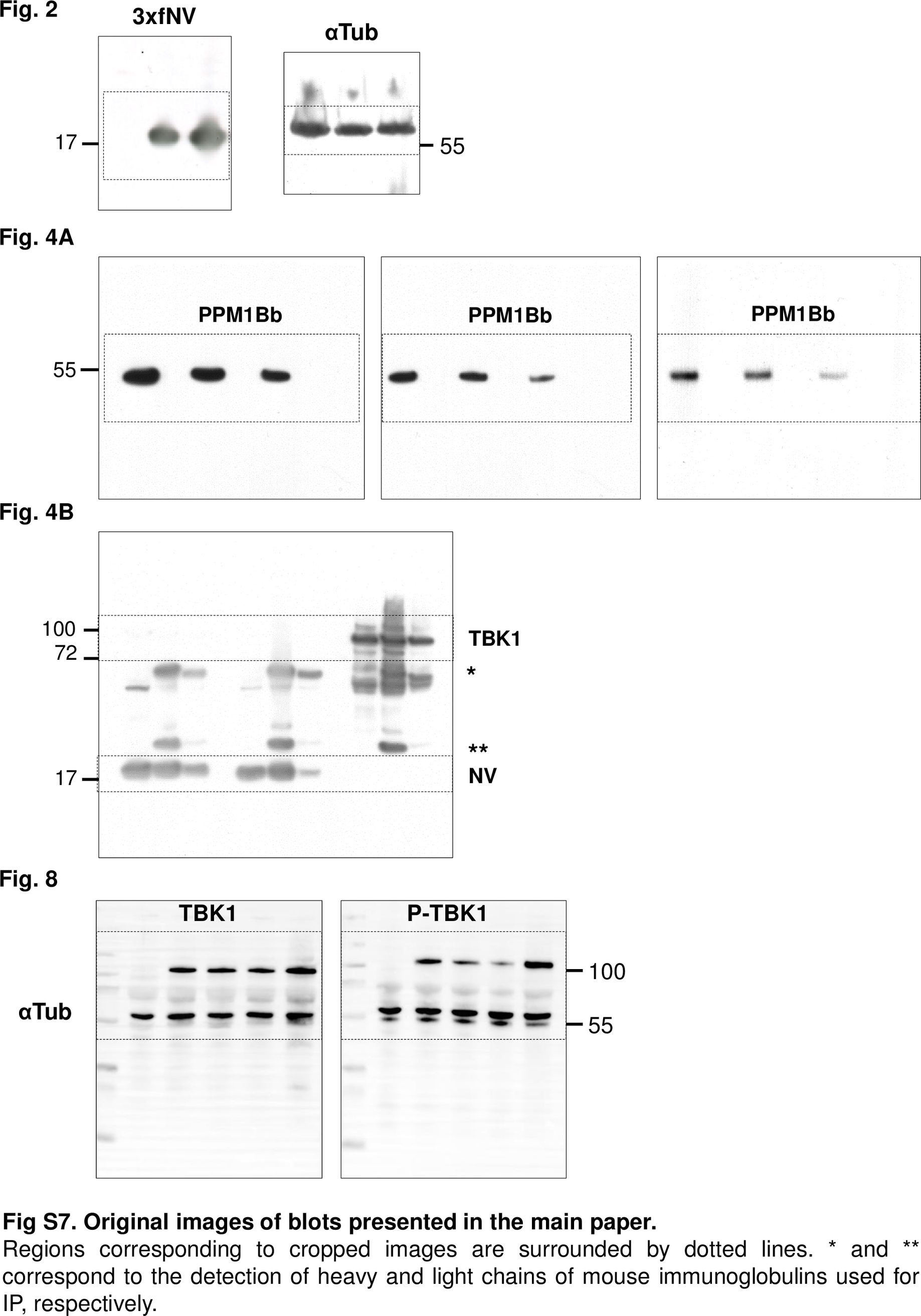

Supplement: Supplementary Information [file srep44025-s1.doc]
